# Alterations in Corneal Sensory Nerves During Homeostasis, Aging, and After Injury in Mice Lacking the Heparan Sulfate Proteoglycan Syndecan-1

**DOI:** 10.1167/iovs.17-21531

**Published:** 2017-10

**Authors:** Sonali Pal-Ghosh, Gauri Tadvalkar, Mary Ann Stepp

**Affiliations:** 1 Department of Anatomy and Regenerative Biology, The George Washington University Medical School, Washington, D.C., United States; 2 Department of Ophthalmology, The George Washington University Medical School, Washington, D.C., United States

**Keywords:** cornea, subbasal nerves, intraepithelial corneal nerves, syndecans, wound healing

## Abstract

**Purpose:**

To determine the impact of the loss of syndecan 1 (SDC1) on intraepithelial corneal nerves (ICNs) during homeostasis, aging, and in response to 1.5-mm trephine and debridement injury.

**Methods:**

Whole-mount corneas are used to quantify ICN density and thickness over time after birth and in response to injury in SDC1-null and wild-type (WT) mice. High-resolution three-dimensional imaging is used to visualize intraepithelial nerve terminals (INTs), axon fragments, and lysosomes in corneal epithelial cells using antibodies against growth associated protein 43 (GAP43), βIII tubulin, and LAMP1. Quantitative PCR was performed to quantify expression of SDC1, SDC2, SDC3, and SDC4 in corneal epithelial mRNA. Phagocytosis was assessed by quantifying internalization of fluorescently labeled 1-μm latex beads.

**Results:**

Intraepithelial corneal nerves innervate the corneas of SDC1-null mice more slowly. At 8 weeks, ICN density is less but thickness is greater. Apically projecting intraepithelial nerve terminals and lysosome-associated membrane glycoprotein 1 (LAMP1) are also reduced in unwounded SDC1-null corneas. Quantitative PCR and immunofluorescence studies show that SDC3 expression and localization are increased in SDC1-null ICNs. Wild-type and SDC1-null corneas lose ICN density and thickness as they age. Recovery of axon density and thickness after trephine but not debridement wounds is slower in SDC1-null corneas compared with WT. Experiments assessing phagocytosis show reduced bead internalization by SDC1-null epithelial cells.

**Conclusions:**

Syndecan-1 deficiency alters ICN morphology and homeostasis during aging, reduces epithelial phagocytosis, and impairs reinnervation after trephine but not debridement injury. These data provide insight into the mechanisms used by sensory nerves to reinnervate after injury.

In vertebrates, the cornea is innervated by a dense collection of sensory nerves with cell bodies in the ophthalmic branch of the trigeminal ganglion. During development, these nerve fibers form rings around the cornea at the developing limbus before migrating to the corneal center in a process dependent on sulfated glycosaminoglycans (GAGs),^1^ Semaphorin 3A, VEGF, and neuropilins.^2–5^ The epithelial surface of the cornea is the most densely innervated surface of the body. The intraepithelial corneal nerves (ICNs) consist of 1 to 40 individual axons bundled together into fibers that range from 0.4 to 5.7 μm in thickness.^6–8^ Subbasal nerves (SBNs) and intraepithelial nerve terminals (INTs) together comprise the ICNs.^9^ En face in vivo confocal imaging visualizes SBNs well but does not allow quantitative assessment of the INTs.^10^ The ICNs form a spiral vortex with axons terminating near the corneal center. Functional studies show that ICNs consist of Aβ and C-fibers, which are capable of sensing temperature and noxious substances.^11–13^ In the stroma, nonmyelinating and myelinating Schwann cells surround the stromal nerves but when nerves leave the stroma and enter the epithelium and become ICNs, Schwann cells remain in the stroma. The ICNs are without glial cell support for distances as long as 5 to 8 mm in large primates including humans.^7^ Corneal epithelial cells provide the ICNs with the support typically supplied by Schwann cells.^9^

We developed a confocal imaging method for whole flat-mounted mouse corneas that allows us to quantify nerve density as a function of time after wounding and have shown in wild-type (WT) mice that ICN density recovers to control levels within 4 days after trephine wounds.^14^ This wound type uses a dulled trephine to crush or sever approximately 50% of the ICNs within a 1.5-mm central circular area; the epithelium within this area remains intact.^14^ Because the trephine does not penetrate the stroma, the wounds do not transect all of the ICNs beneath the trephine blade. Regeneration of vortex and ICN density after 1.5-mm trephine wounds occurs in WT mice within several days. By contrast, after 1.5-mm debridement wounds where epithelial cells and ICNs are removed, corneas fail to fully regenerate their ICNs, and develop recurrent epithelial erosions.^15,16^

The heparan sulfate (HS) glycosaminoglycan chains present on heparan sulfate proteoglycans (HSPGs) have been proposed to serve as a “sugar code” for vertebrate neuronal development by regulating signaling.^17^ Deletion of enzymes required for formation of HS side chains, removal of chains with the enzyme heparanase, and/or injection of HS into developing vertebrate embryos alter axon guidance during development.^17,18^ Heparan sulfate proteoglycans act cell-autonomously as receptors, by clustering with other receptors within specific membrane domains, or by regulating membrane trafficking during endocytosis, exocytosis, and exosome formation.^19^ Heparan sulfate proteoglycans also function noncell-autonomously by modifying extracellular matrix composition and creating and maintaining gradients of signaling molecules. An essential role for HS in corneal epithelial cell homeostasis has been demonstrated by studies performed in genetically engineered mice that lack expression of HS in corneal epithelial cells.^19,20^

Axonal recovery after injury requires the coordination of numerous events.^21,22^ Nerve cell bodies must detect the injury and change their gene expression; reinnervating axons must interact with supporting cells, typically inflammatory and glial cells, form growth cones, and extend through a complex extracellular matrix to target sites. Regeneration-associated genes (RAGs) produced by neurons regulate axon extension in the central nervous system and peripheral nervous system; functions of several RAGs are mediated by HS binding.^23–26^ In response to debridement injuries to the WT mouse cornea, the mRNAs for several HS-regulated RAGs including *DCC*, *EFNA4*, and *EFNA5* increase, whereas expression of Netrin (*NTN*) drops suggesting that differences in the interaction between corneal epithelial cells and HS-binding RAGs accompany corneal wound healing and reinnervation.^14^

Syndecans (SDCs) are single pass integral membrane HSPGs.^27,28^ C. elegans and Drosophila express a single SDC, whereas vertebrates have four SDC homologs: SDC1, SDC2, SDC3, and SDC4. Syndecans 1 has been studied most in epithelial cells where it is highly expressed, whereas SDC3 is better characterized in the nervous system where it is expressed on axons and Schwann cells. Mice lacking SDC1 or SDC3 are viable and studies have shown both adverse (loss of function) and beneficial (gain of function) effects depend on the cells and tissues affected by the loss of the proteoglycan.^29–31^

A recent study by Edwards and Hammarlund^32^ in C. elegans shows that when SDC is inactivated, axon regeneration and growth cone guidance are impaired. The growth cones of axons lacking SDC are less stable; the ability to stabilize growth cones is mediated by the SDC core protein expressed on neurons and by HS chains located on nonneuronal cells and tissues. Studies in drosophila confirm the importance of SDC in axon growth cones and also show that both extracellular and cytoplasmic domains are required for proper synapse formation.^33^

Syndecan 1-null mice on the BALB/c background have transparent corneas that remain clear as the mice age and after 1.5-mm corneal debridement wounds. Corneal wound healing studies show that SDC1-null mice heal debridement wounds significantly slower than WT mice.^34^ After re-epithelialization is complete, there is a significant reduction in the frequency with which their corneas develop spontaneous recurrent epithelial erosions.^16^ Syndecan 1-null keratinocytes are more adhesive than WT keratinocytes due to enhanced activity of αv-family integrins and altered TGFβ1 signaling.^29^ As a result, they assemble an ECM that differs from that produced by WT keratinocytes. Sensory nerves are stabilized within epithelial tissues by adhering via integrins to ECM proteins produced by supporting cells.^35–37^ Because SDC1-null corneal epithelial cells are more adhesive, adhesion between them, and ICNs may impact their morphology and their reinnervation after injury. Altered ECM assembly may modify the ECM around reinnervated axons. Integrins including αvβ5 regulate the ability of cells to phagocytose extracellular debris^38,39^ and degrade it within lysosomes.^40^ Because αv-integrin activity is enhanced in multiple cell types derived from the SDC1-null mice including epithelial cells,^29,41,42^ altered phagocytosis of shed intraepithelial nerve terminals by SDC1-null corneal epithelial cells may occur. How these differences impact ICN morphology and reinnervation after injury is not known.

Small fiber neuropathy is increasing in our aging population and can cause itching, burning, cold, and/or prickling sensations as well as loss of sensation in the skin. A diagnosis of small fiber neuropathy is confirmed by quantifying nerve fibers in skin biopsies using sensory nerve markers including βIII tubulin and growth associated protein 43 (GAP43). Small fiber neuropathy is associated with reduced sensory axon density^43^ and loss of GAP43 expression in the skin.^44^ GAP43 is expressed within sensory axons in the cornea^45^ and has been studied in the mouse cornea at the mRNA level before and after injury.^46^ Patients with Sjogren's syndrome have small fiber neuropathy at various sites in their bodies^47,48^ including corneal ICNs.^49,50^ In 2009, a study showed that in vivo confocal imaging could be used to confirm diagnosis of Fabry disease.^51^ Since then, more than 20 studies have been reported using in vivo confocal imaging of the cornea to diagnose small fiber neuropathy due to various causes. Despite limitations, clinicians will likely continue to increase their use of this noninvasive assessment tool for diagnosis and evaluation of progression of small fiber neuropathy.^10^

To gain insight into the roles played by the ICNs in mediating repair of the ocular surface after injury, we assess the nerve density and axon thickness in SDC1-null mice as a function of time after birth through 10 months of age. We also evaluate changes in nerve density and thickness over time after 1.5-mm debridement and 1.5-mm trephine injury, where 50% of the ICNs are degraded in response to rotating a centrally placed dulled trephine that crushes and/or severs ICNs but leaves the epithelium in place. For the trephine-injured corneas, we also localize βIII tubulin, GAP43, and the lysosomal marker lysosome-associated membrane glycoprotein 1 (LAMP1) in INTs. In WT and SDC1-null corneal epithelial cells and epidermal keratinocytes, we assess phagocytosis. Taken together, these studies provide new insight into the mechanisms used by HSPGs to mediate PNS sensory nerve homeostasis and reinnervation and highlight the importance of debris clearance in ICN regeneration after injury.

## Materials and Methods

### Animals

All studies performed comply with The George Washington University Medical Center Institutional Animal Care and Use Committee guidelines and with the ARVO Statement for the Use of Animals in Ophthalmic and Vision Research. For all wounding experiments, 7- to 8-week male and female (virgin) mice were used. The mouse SDC1 gene structure was described previously.^52^ The SDC1-null mice were bred in house at The George Washington University Animal Facility. BALB/c mice were ordered from Charles River (Frederick, MD, USA). For developmental studies, mice were euthanized at 2, 3, 4, 5, 8, 14, and 16 weeks. For 4, 8, and 16 weeks, both male and female mice were studied. For wounding studies, mice were anesthetized with ketamine/xylazine and a topical anesthetic applied to their ocular surface. For trephine wounds, a 1.5-mm dulled trephine was used to demarcate the wound area. For debridement wounds, the epithelial cells within a 1.5-mm trephine area were removed using a dulled blade. Wounding was bilateral for each wound type. After wounding, erythromycin ophthalmic ointment was applied to the injured cornea and mice were allowed to heal for 1, 2, 3, 4, 7, 14, and 28 days, then euthanized. For trephine studies, an additional time point of 42 days was assessed. The number of mice used for each developmental and injury time point is shown in Table 1; images of individual corneas are provided in Supplementary Data. When data show a significant difference in axon density between genotypes in male mice, experiments are repeated using WT and SDC1-null female mice to determine whether their sex contributed to the differences observed. The axon density data presented for WT mice after debridement and trephine injury have been published previously.^14^ They are included here to permit us to make quantitative comparisons between WT and SDC1-null axon densities after the same types of injury. For immunofluorescence (IF) studies, tissues were pooled for each time point and wound type and fixed immediately after euthanization in a paraformaldehyde-containing fixative (1x PBS, 1% formaldehyde, 2-mM MgCl_2_, 5-mM EGTA, 0.02% NP-40) for 1 hour and 15 minutes at 4°C, followed by two washes for 10 minutes each in 1x PBS containing 0.02% NP40 at room temperature. Tissues were placed in 4:1 methanol:dimethyl sulfoxide (DMSO) for 2 hours at −20°C and then stored in 100% methanol at −20°C until used for whole-mount staining studies.

**Table 1:**
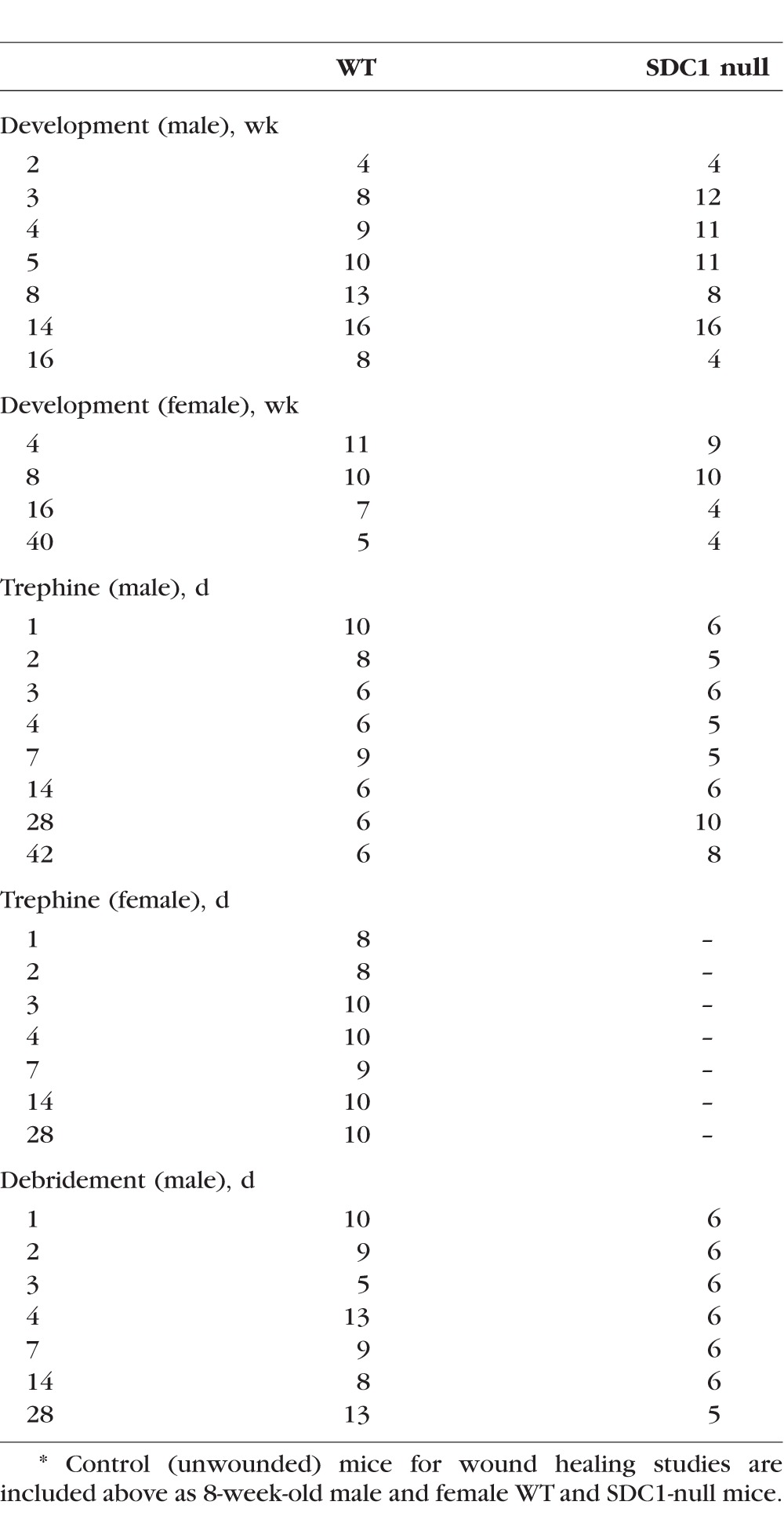
Number of Corneas Used for Sholl and Axon Thickness Analyses*

**Figure 1:**
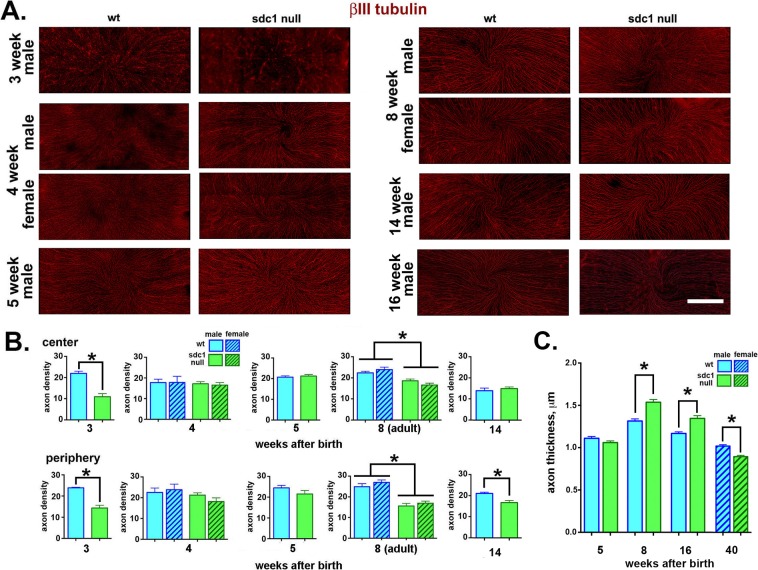
After birth, SDC1-null corneas acquire ICNs slower, organize them into thicker bundles, and retain ICN thickness longer than WT mice. (A) Representative images obtained from male and female WT and SDC1-null mouse corneas at various times after birth are presented. The ICNs have been visualized using an antibody against βIII tubulin. Male and female corneas are shown at 4 and 8 weeks after birth; male corneas are shown at 3, 5, 14, and 16 weeks after birth. (B) Images including those presented in (A) were used to quantify the axon density of the ICNs using Sholl analysis. Data indicate that axons are significantly less dense at the center and periphery of the 3-week SDC1-null mouse corneas compared with WT corneas. Axon density in SDC1-null corneas increases to levels similar to WT at 4 and 5 weeks. By 8 weeks (adult), the axon density in SDC1-null corneas at the center and periphery is less than that seen in WT corneas. No differences in axon density are seen in male and female corneas at 4 and 8 weeks. By 14 weeks, ICN density is similar in the center for both genotypes but remains lower in the periphery in SDC1-null corneas. Intraepithelial corneal nerves in 16-week WT male corneas could not be analyzed due to increased background staining and reduced axon thickness. (C) Axon thickness measurements for unwounded corneas are similar at the center and periphery so data are combined and the mean thickness of 100 axons per WT and SDC1-null corneas in male mice at 5, 8, and 16 weeks and for female mice at 40 weeks were studied. Data show that ICNs in WT and SDC1-null corneas are similar in thickness at 5 weeks of age. By 8 and 16 weeks, SDC1-null ICNs are significantly thicker than WT ICNs. However, by 40 weeks of age, ICN thickness is significantly lower in SDC1-null female corneas. Note that ICN thickness is maximal for both genotypes at 8 weeks of age. Scale bar in (A): 250 μm.

**Figure 2:**
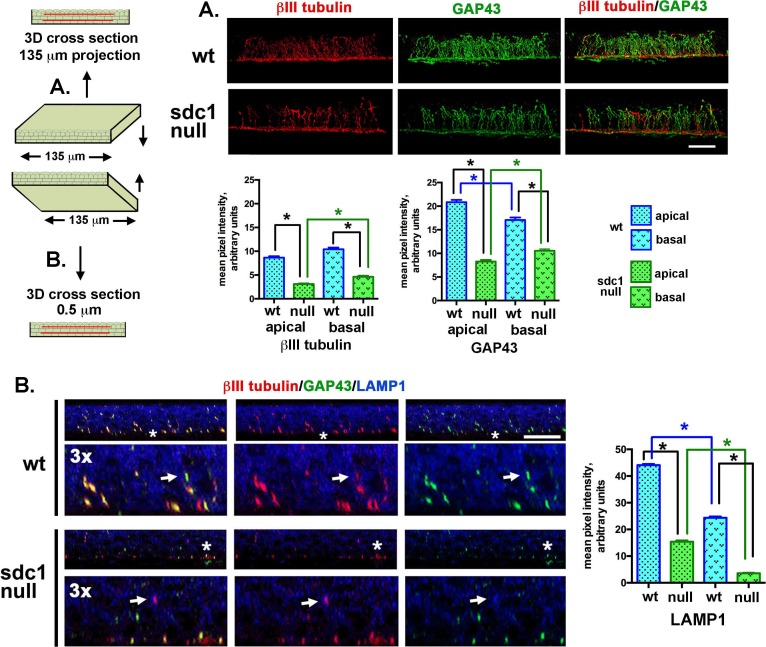
Differences in INTs and LAMP1 localization in WT and SDC1-null adult corneas. Representative high-resolution confocal 3D images rotated as indicated in the cartoon to the left were used to generate cross sectional views to show the localization of βIII tubulin (red) and GAP43 (green) in intraepithelial nerve terminals in control WT and SDC1-null corneas. The images shown in (A) project through 135 μm of tissue, images in (B) project through 0.5 μm of tissue. (A) Intraepithelial corneal nerves are shown to be linear, arise from the subbasal nerves (SBNs), and project apically. Quantitation of βIII tubulin and GAP43 within apical and basal layers of the corneal epithelium reveals that WT corneas have significantly more INTs in both basal and apical cell layers compared with SDC1-null corneas. Although SDC1-null corneas have fewer INTs, they are equally capable of extending toward the apical most cell layers. (B) In the 0.5-μm cross-sectional slices through the tissue, INTs appear discontinuous. In addition to βIII tubulin and GAP43, LAMP1 is shown in blue. LAMP1 is a lysosomal marker; co-localization of GAP43 and/or βIII tubulin within corneal epithelial cell lysosomes indicate that axon fragments have been phagocytozed and are being degraded (arrows). The sites indicated by the asterisks have been digitally enlarged 3-fold. LAMP1 was quantified in WT and SDC1-null corneas. Data show that cells within the apical layers express more LAMP1 compared with basal cells in both genotypes of mice. In addition, WT corneas express 2- to 3-fold more LAMP1 in apical and basal layers compared with SDC1-null corneas. Scale bar: 10 μm.

**Figure 3:**
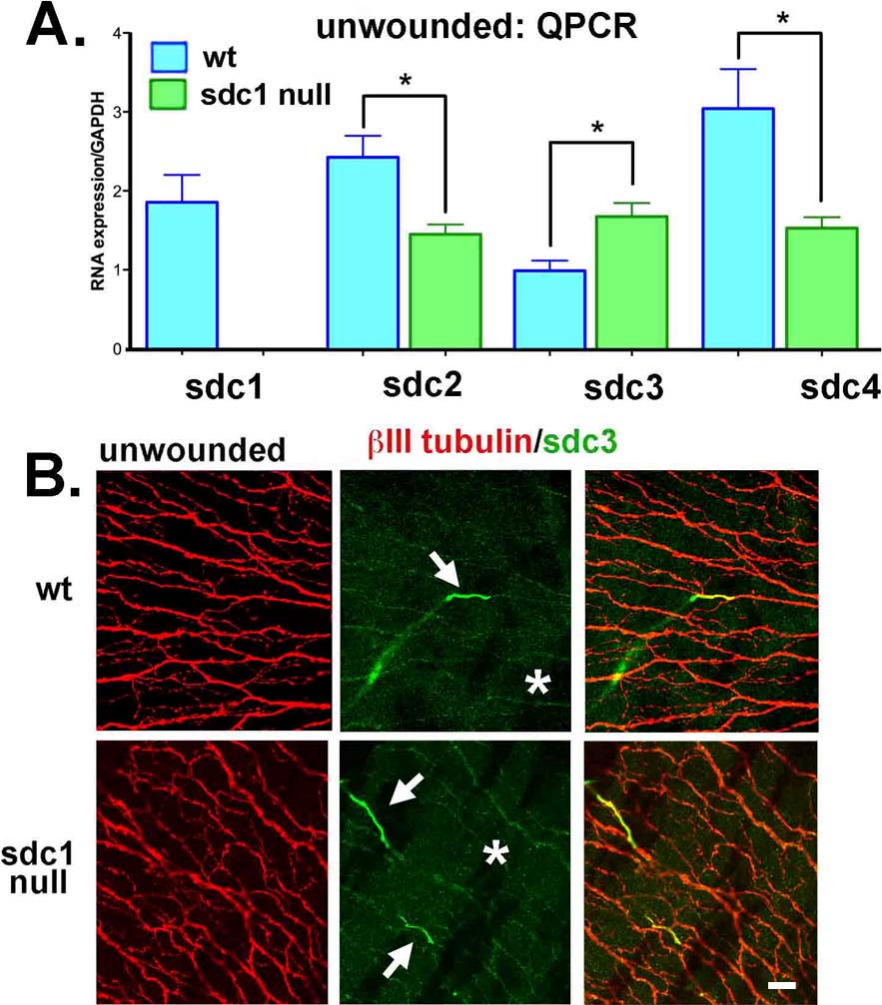
Loss of SDC1 leads to reduced mRNA expression for SDC2 and SDC4 and increased expression of SDC3 mRNA and protein within SDC1-null ICNs. (A) Quantitative PCR was performed using primers for SDC1, SDC2, SDC3, and SDC4 on RNA extracts obtained by limbal to limbal debridement from unwounded 7- to 9-week-old WT and SDC1-null corneas. Data show that RNA for SDC2 and SDC4 are significantly reduced in SDC1-null mice, whereas SDC3 is increased. (B) Because SDC3 is expressed in the CNS and PNS, we next stained corneas with antibodies against SDC3 (green) and βIII tubulin (red). In WT and SDC1-null corneas, SDC3 is localized on short segments of the stromal nerves as they exit the epithelium and become ICNs (*). In SDC1-null corneas, numerous ICNs show faint but detectible expression of SDC3 compared with WT corneas. Scale bar: 10 μm.

**Figure 4:**
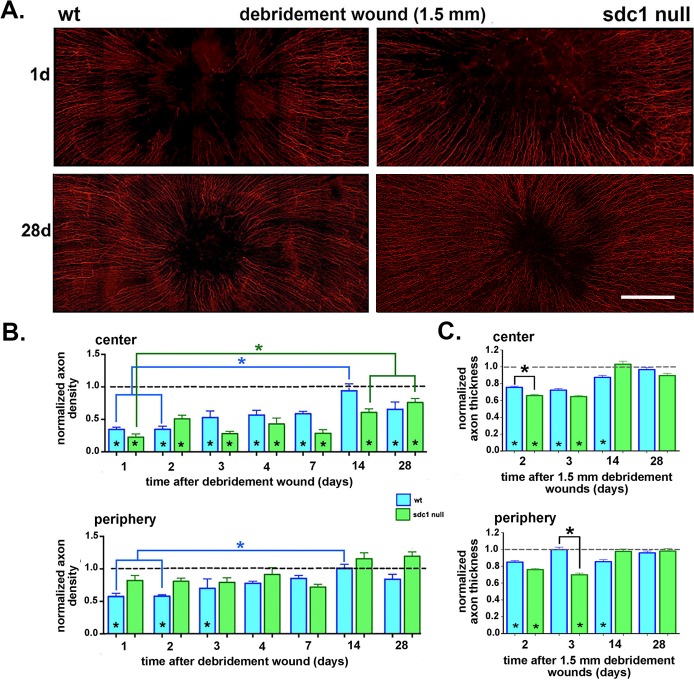
Axon density improves at similar rates at the center of SDC1-null and WT corneas after 1.5-mm debridement wounds. (A) Corneas from WT and SDC1-null mice injured by 1.5-mm debridement wounding are stained with an antibody against βIII tubulin to visualize the ICNs. Representative images from adult male WT and SDC1-null mouse corneas at 1 and 28 days after debridement wounding are shown. (B) Images including those presented in (A) were used to quantify the axon density of the ICNs using Sholl analysis at 1, 2, 3, 4, 7, 14, and 28 days after debridement. Data are normalized relative to unwounded controls for each genotype. Asterisks within bars indicate significant differences in axon density relative to unwounded controls. (C) Axon thickness was assessed at the center and periphery 2, 3, 14, and 28 days after debridement wounding in WT and SDC1-null corneas. Data have been normalized relative to unwounded mice. Asterisks within bars indicate significant differences in axon thickness relative to unwounded controls. Data show that normalized axon thickness decreases significantly relative to controls at 2 and 3 days in the center of SDC1-null corneas and at 2, 3, 14 days in the center of WT corneas. At the periphery in SDC1-null mice, normalized axon thickness is similar to controls at 14 and 28 days but lower than controls at 2 and 3 days. At the periphery in WT mice, normalized axon thickness is lower than control at 2 and 14 days but similar to controls at 3 and 28 days. Scale bar in (A): 250 μm.

**Figure 5:**
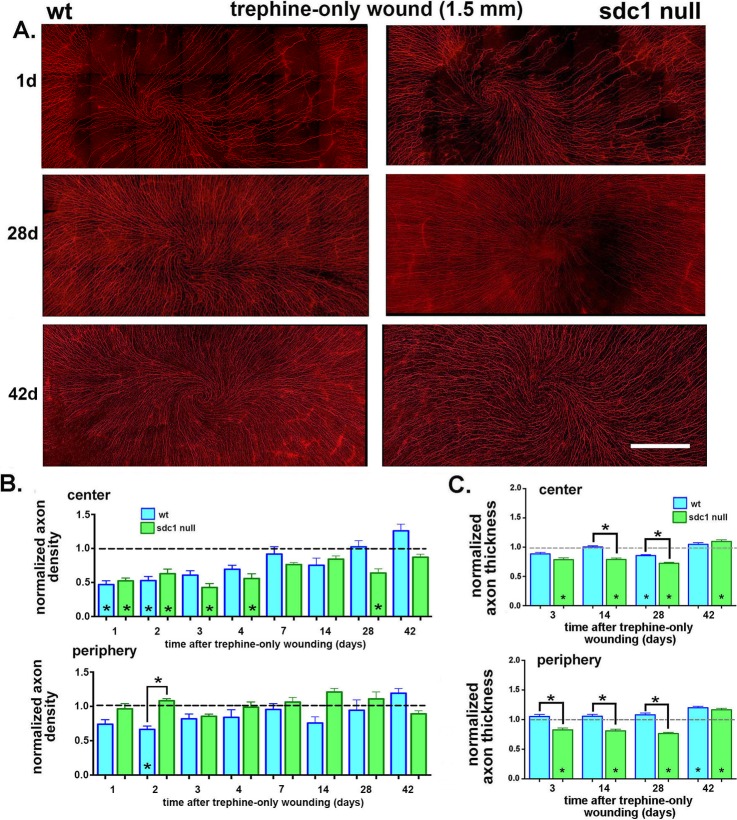
Syndecan-1–null axons are impaired in their ability to reinnervate the cornea after trephine injury. (A) Corneas from WT and SDC1-null mice were obtained at the following times after trephine injury: 1, 2, 3, 4, 7, 14, 28, and 42 days. Axons were visualized using an antibody against βIII tubulin. Representative images obtained 1, 28, and 42 days after trephine injury are shown. Both genotypes appear to lose similar numbers of ICNs after trephine injury at 1 day. Syndecan-1–null corneas continue to improve between 28 and 42 days; WT corneas appear similar to controls at 28 days. (B) Images of corneas stained for ICNs were used to quantify the axon density using Sholl analysis. Data are normalized relative to 8-week unwounded controls for each genotype with the exception of the 42-day injured corneas, which were normalized against genotype and age-matched controls to compensate for the loss of axon density with aging. Asterisks within bars indicate significant differences in axon density relative to unwounded controls. In the corneal center, normalized WT ICN density is restored to control levels at 3 days; SDC1-null ICN density is restored to control levels by 7 days but reduces again at 28 days before increasing at 42 days. (C) Axon thickness was assessed at the center and periphery at 3, 14, 28, and 42 days after trephination in WT and SDC1-null corneas. Data have been normalized relative to unwounded mice. Normalized axon thickness is reduced in SDC1-null corneas compared with controls at 3, 14, 28, and 42 days in the corneal center and periphery. It is reduced compared with WT in the center at 14 and 28 days and in the periphery at 3, 14, and 28 days. Scale bar in (A): 300 μm.

For the whole-mount procedure, the back of the eye was cut, retina, lens, and iris removed before staining. Tissues were transferred to a graded Methanol-TritonX-100 series (75%, 50%, and 25% methanol:TritonX-100 for 15, 15, and 10 minutes, respectively). All incubations were performed with gentle shaking and at room temperature, unless otherwise specified. The eyes were washed twice in PBS, for 30 minutes each, followed by incubation with blocking buffer for 2 hours. Blocking buffer was made as follows: to 100 mL 1x PBS, 1 g of BSA was added, the mixture was stirred for 10 minutes, 1 mL of horse serum was added, and the mixture was stirred for an additional minute. The tissues were then incubated overnight with primary antibody diluted in blocking buffer at 4°C. The next day, the tissues were washed five times with PBS and 0.02% Tween 20 (PBST) for 1 hour each, blocked for 2 hours, and then incubated with secondary antibody diluted in blocking buffer overnight at 4°C. The following day, eyes were washed three times with PBST for 1 hour each, followed by nuclear staining with 4′,6-diamidino-2-phenylindole (DAPI) for 5 minutes, and washed with distilled water.

### Antibodies

Corneas were stained with the following antibodies: βIII tubulin (#801201; Biolegend, San Diego, CA, USA), GAP43 (#NB300-143; Novus Biological, Littleton, CO, USA), mLAMP1 (#AF4320; R&D Systems, Minneapolis, MN, USA), and SDC3 (#SC9496; Santa Cruz Biotechnology, Dallas, TX, USA). Appropriate secondary DyLite 488, 594, and 647 antibodies from Jackson Immunobiologicals (West Grove, PA, USA) were used for immunolabeling. Corneas were stained with DAPI (#46190; Thermo Fisher Scientific, Grand Island, NY, USA) before flat mounting to visualize nuclei. To achieve the best flattening, the corneas were placed epithelial side-up with mounting media (#17984-25, Fluoromount G; Electron Microscopy Sciences; Hatfield, PA, USA) and coverslipped.

### Microscopy

Confocal microscopy was performed at the GW Nanofabrication and Imaging Center at The George Washington University Medical Center. For Sholl analysis, images were acquired using the Zeiss Cell Observer Z1 spinning disk confocal microscope (Carl Zeiss, Inc., Thornwood, NY, USA), equipped with ASI MS-2000 (Applied Scientific Instrumentation, Eugene, OR, USA) scanning stage with z-galvo motor, and Yokogawa CSU-X1 spinning disk (Yokogawa, Sugar Land, Texas, USA). A multi-immersion 25×/0.8 objective lens, LCI Plan-Neofluor (Carl Zeiss, Inc.), was used for imaging, with oil immersion. Evolve Delta (Photometrics, Tucson, AZ, USA) 512 × 512 EM-CCD camera was used as detector (80-msec exposure time). A diode laser emitting at 568 nm was used for excitation (54% power). Zen Blue software (Carl Zeiss, Inc.) was used to acquire the images, fuse the adjacent tiles, and produce maximum intensity projections. The adjacent image tiles were captured with overlap to ensure proper tiling. All images were acquired using the same intensity settings. Sholl analysis was performed using ImageJ software (http://imagej.nih.gov/ij/; provided in the public domain by the National Institutes of Health, Bethesda, MD, USA) as described previously.^14^ Panels including images of corneas for all time points assessed by Sholl analysis are included in Supplementary Figures S1 through S9. Images have been reduced in size and resolution.

For high-resolution IF imaging, a confocal laser-scanning microscope (Zeiss 710; Carl Zeiss, Inc.) was used to image the localization of Alexa Fluor 488 (argon laser; 488-nm laser line excitation; 495/562 emission filter; Jackson Immunobiologicals), Alexa Fluor 594 (561 diode laser; 594-nm nm laser line excitation; 601/649 emission filter; Jackson Immunobiologicals), and Alexa Fluor 647 (633 Diode laser; 647-nm laser line excitation; 671/759 emission filter; Jackson Immunobiologicals). Optical sections (*z* = 0.5 or 1 μm) were acquired sequentially with a 63× objective lens. Three-dimensional (3D) images were rotated to generate cross section views using Volocity software (Version 6.3; Perkin Elmer, New York, NY, USA). High-resolution images are presented either as cross sections projected through the length of the acquired image (135 μm), or as cross-sections projected 0.5 μm of tissue.

**Figure 6:**
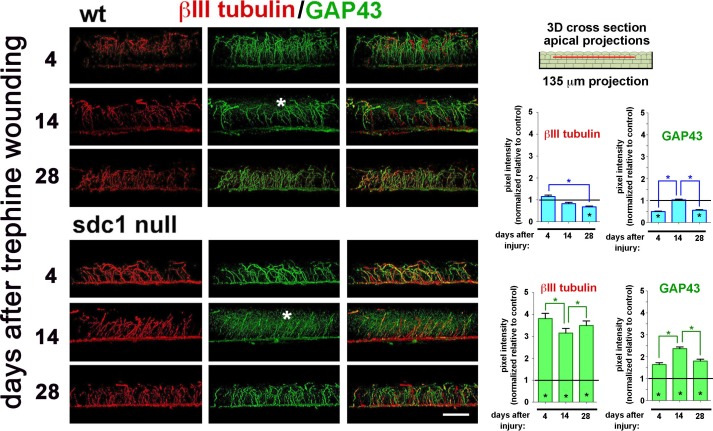
Intraepithelial nerve terminals extension into the apical most cell layers increases in SDC1-null corneas after trephine injury. Representative 3D images rotated to generate cross-sectional views show the localization of βIII tubulin (red) and GAP43 (green) at 4, 14, and 28 days after trephine wounding in WT and SDC1-null corneas. Punctate GAP43 is increased within the epithelium in SDC1-null compared with WT corneas at 14 days (*). βIII tubulin+ and GAP43+ INTs in the apical most cell layers were quantified as a function of time after trephine injury and normalized relative to control (presented in Fig. 2A). Asterisks within bars indicate differences that are significant relative to unwounded controls; asterisks between bars indicate significant differences between time points. In WT corneas, the numbers of βIII tubulin+ and GAP43+ INTs extending apically decrease relative to controls at 28 days for βIII tubulin+ INTs and at 4 and 28 days for GAP43+ INTS, in SDC1-null corneas, both βIII tubulin+ and GAP43+ INTs increase at all time points assessed. Scale bar: 25 μm.

**Figure 7:**
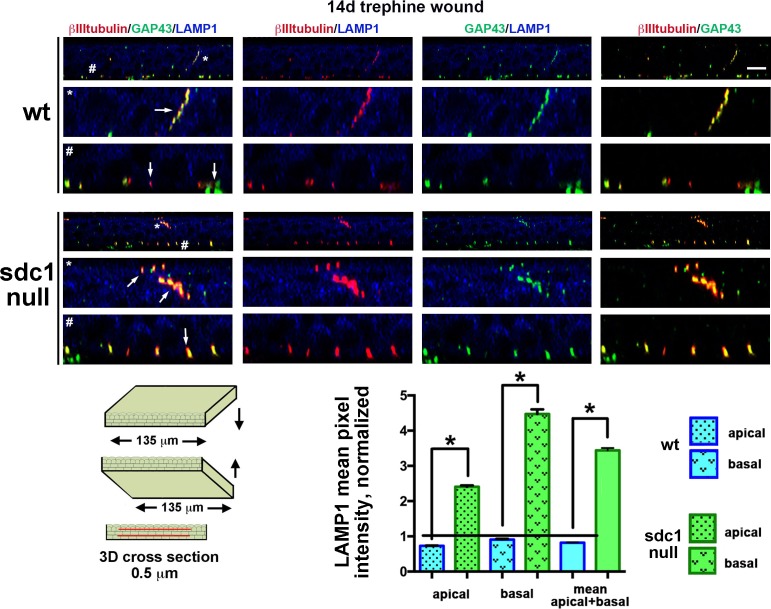
LAMP1 increases in SDC-null corneas 14 days after trephine wound. Representative 3D images rotated to generate cross-sectional views show the localization of GAP43 (green), βIII tubulin (red), and LAMP1 (blue) in WT (top) and SDC1-null (bottom) corneas. These images show staining within a 0.5-μm cross-sections. Data indicate that axon fragments are being degraded (arrows). Representative INTs (*) and SBNs (#) have been highlighted and enlarged 3-fold. When LAMP1 expression within apical and basal cell layers 14 days after trephine injury is quantified and compared with unwounded controls (Fig. 2B), LAMP1 increases over 3-fold in SDC1-null corneas but remains the same in WT corneas. Scale bar: 10 μm.

### Mouse Corneal Epithelial and Keratinocyte Phagocytosis Studies

For explant cultures, a dulled 1.5-mm trephine was used to demarcate the wound area and epithelial tissues within the area removed using a dulled blade as described above. After wounding, erythromycin ophthalmic ointment was applied to the injured cornea and allowed to heal for 18 hours after which the mice were euthanized. Corneal buttons were dissected free of limbal and conjunctival tissues and cuts were made to flatten the cornea. Corneas were placed endothelial side down on 6-well plates that had been previously coated with fibronectin/collagen I (FN/CN) (37°C for 30 minutes).^29^ Tissue culture plates were placed in the incubator for 60 minutes with the explants. Keratinocyte serum free medium (KSFM) (#17005-042; Invitrogen, Carlsbad, CA, USA) was added to the plate to cover the explant surface. For these studies KSFM was prepared as follows: 1.25-mL bovine pituitary extract, 4-μL epidermal growth factor, and 5-mL pen-strep solution (#15140-122; Gibco, Gaithersburg, MD, USA) were added to each 500-mL bottle of KSFM. Explants were fed with KSFM every 48 hours. Primary mouse keratinocytes were obtained from WT and SDC1-null neonatal pups and cultured as described previously.^29^

For the phagocytosis assays, FluoSphere sulphate, 1.0-μm, red (580/605) beads, (#1850395; Invitrogen) were used. Before use, approximately 100 μL of packed bead volume was suspended in 300 μL of 1% fetal bovine serum (FBS) in PBS and allowed to opsonize serum proteins for 2 hours at 37°C; beads were vortexed every 15 minutes during opsonization followed by centrifugation at 1620*g* for 5 minutes and washed three times with PBS. The washed and opsonized beads (100 μL) were resuspended in 8 mL of the appropriate media.

For explants, beads were added to the cultures at 3 days and incubated overnight. Two explants were assessed per genotype and the experiments were repeated allowing beads to incubate with explants for 1 hour and the results obtained both times were similar.

For mouse keratinocytes, WT and SDC1-null cells, 3 days after being placed in culture, were incubated in media containing opsonized beads for 1 hour. Phagocytosis was inhibited in keratinocyte cultures by addition of two inhibitors to the media the same time the beads were added: Chloroquine (CQ; #C6628-25G, used at 25 μm; Sigma Aldrich, St. Louis, MO, USA) and Bafilomycin A1 (BafA1; #B1793-2UG, used at 200 nm, stock made up in DMSO; Sigma). After incubation with beads, explants and cells were washed 3 times with PBS and then fixed in 4% paraformaldehyde (#28906; Thermofisher Scientific) in PBS for 15 minutes at RT. Following fixation, cells were stained with phalloidin (#12379; Invitrogen). After confirming that beads were internalized within cells by assessing phalloidin staining, 10× fluorescent and bright field images were obtained with an Olympus IX81 scope (Center Valley, PA, USA) and overlaid using Metamorph Basic Version 7.7.3.0 (Sunnyvale, CA, USA) to allow quantitative assessment of the numbers of beads internalized.

### Quantification of Fluorescence Intensity

ImageJ was used to quantify the data. Confocal images (63× 3D) were generated using Volocity (Version 6.3; Perkin Elmer, New York, NY, USA) and rotated to generate cross sections as shown in the cartoons. Each image subjected to quantification was obtained using the same confocal laser settings and the same intensity settings in Volocity to permit valid comparisons. Two representative images were selected for each variable and subjected to quantification. Each image to be quantified was opened in ImageJ and magnified to 150%. For INT quantification, two lines were drawn parallel to the SBNs. One line was just above the SBNs and located basally and the second was located apically. For INT quantification after wounding, a single apical line was drawn. Mean gray pixel intensity values were obtained for each image using Plot Profile function of ImageJ. For quantification of LAMP1 intensity, a similar approach was taken to that used for Figure 2A.

**Figure 8:**
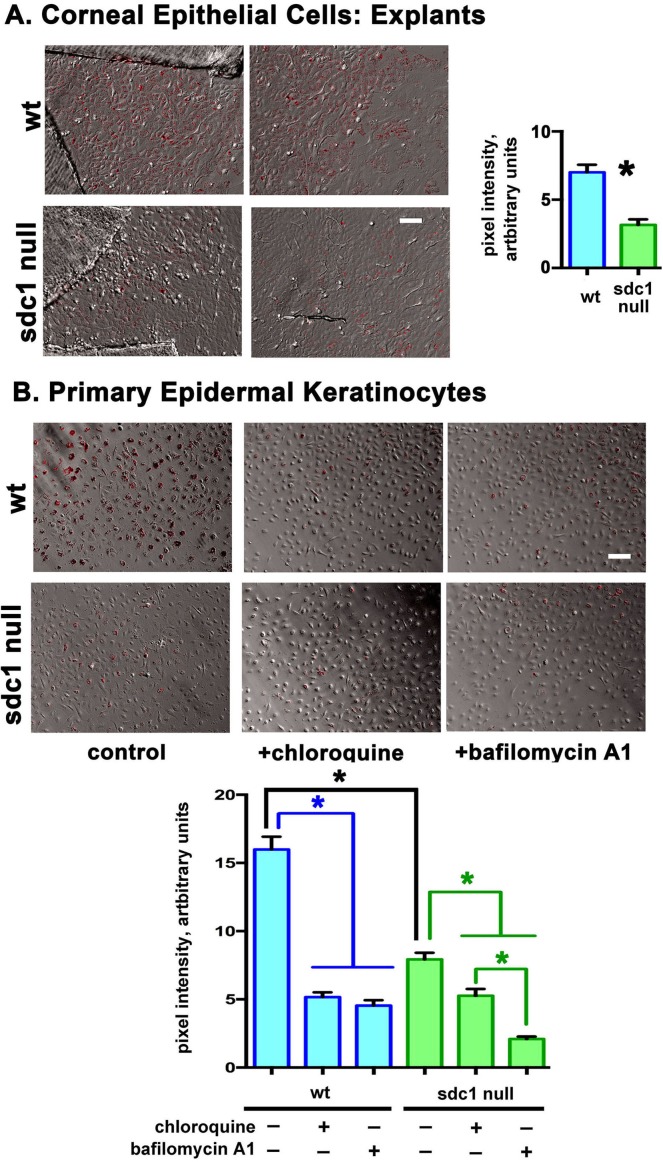
Loss of SDC1 impairs the ability of SDC1-null corneal epithelial cells and epidermal keratinocytes to phagocytose opsinized beads. Explant-derived corneal epithelial cell cultures were obtained from adult WT and SDC1-null mouse corneas and the ability of the cells to phagocytose 1-μm opsinized fluorescent latex beads were compared. SDC1-null corneal epithelial cells phagocytosed 50% fewer beads compared with WT cells. SDC1-null epidermal keratinocytes showed similar phenotypes. Two well characterized inhibitors of phagocytosis (CQ and BafA1) significantly reduce bead uptake in both genotypes of cells although CQ suppress bead up-take more in WT cells than in SDC1-null cells. Scale bar: 20 μm.

For phagocytosis assays, brightfield and fluorescent images were taken at 10× for both explants and keratinocytes. Representative images from two explants for each genotype were subjected to quantification. For epidermal keratinocytes, representative images from 3-wells per variable were obtained for WT and SDC1-null cells and of cells treated with CQ and BafA1 to inhibit phagocytosis. The gray scale pixel intensity values were obtained for areas within 20-μm circles of 50 randomly selected cells per image with a minimum of 2-wells per variable.

### Quantitative PCR (qPCR)

For qPCR studies, epithelium was scraped using a dulled blade and frozen immediately in liquid nitrogen. Epithelium from six corneas was pooled into a single sample; eight samples were assessed per genotype. RNA was extracted from corneal epithelium using Arcturus Picopure RNA isolation kit (#12204-01; Applied Biosystems, Foster City, CA, USA) according to the manufacturer's instructions. Quantitative PCR was performed using a Bio-Rad CFX384 Real-Time PCR detection system (Hercules, CA, USA). The primers used were ordered from Qiagen (Germaintown, MD, USA), unless otherwise specified: SDC1 (#QT00106288), SDC2 (#QT00144424), SDC3 (#QT00146244), SDC4 (#QT00102130), and GAPDH (267553E06; Invitrogen). Quantitative PCR data is normalized against GAPDH.

### Statistical Analyses

Quantitative data are presented as mean ± SEM. All data were analyzed using 1-way ANOVA. All statistical tests were performed using the GraphPad Prism Program, Version 6 (GraphPad Software, Inc. San Diego, CA, USA). A *P* value less than 0.05 was considered statistically significant.

## Results

### ICN Innervation is Delayed After Birth in SDC1-Null Mice and Differences in ICN Morphology Persist in Adult Mice

To determine whether loss of SDC1 impacts the microanatomy of the ICNs, WT and SDC1-null corneas were stained at 2, 3, 4, 5, 8, 14, and 16 weeks after birth with an antibody against the βIII isoform of tubulin, an isoform expressed abundantly in axons. The numbers of corneas used for each variable assessed are indicated in Table 1. Sexes of the 2-week pups were not assessed; we could not visualize ICNs in WT and SDC1-null corneas 2 weeks after birth regardless of whether the eyelids of the pups were open or closed (data not shown). The axon densities of the ICNs in corneas from male mice were assessed by Sholl analysis at 3, 4, 5, 8, 14, and 16 weeks; axon density in male and female corneas were assessed at 4 and 8 weeks. Representative images are shown in Figure 1A. Panels showing images of each cornea assessed are presented in Supplementary Figures S1 through S3 and S6; data for 14-week (42-day) controls are included in Supplementary Figure S5 for WT and Supplementary Figure S7 for SDC1-null mice. Quantification of axon densities in the center and periphery are shown in Figure 1B.

By 3 weeks after birth, numerous ICNs are present in SDC1-null mice but the vortex cannot be seen. In WT mice, at 3 weeks, a denser collection of ICNs is observed. While a fully formed vortex is not observed, evidence of initial vortex formation can be seen. Sholl analysis indicates that axon density in SDC1-null mice at 3 weeks is significantly lower (50%–55%) at both the center and periphery compared with WT corneas. By 4 weeks, the vortex is formed and no significant differences in axon density are seen between genotypes and sexes. At 5 weeks, no genotype or anatomical (central verses peripheral) differences in axon density are observed; 10 of 10 WT corneas and 10 of 11 SDC1-null corneas have a vortex. By contrast, at 8 weeks, axon density in SDC1-null mice is significantly less than that of WT at both the center (17%) and periphery (37%). Decreased axon density in 8-week SDC1-null corneas is accompanied with the loss of a detectable vortex in 25% (*n* = 8) of male and 30% (*n* = 10) of female corneas (Supplementary Fig. S2). None of the WT 8-week male (*n* = 13) or female (*n* = 10) corneas lack a vortex. These differences in the ICN vortex persist in 14-week-old WT (Supplementary Fig. S5) and SDC1-null (Supplementary Fig. S7) corneas. Axon density at 14 weeks in WT mice decreases compared with 8 weeks at both the center (41%) and periphery (19.3%). In 14-week SDC1-null male mice axon density also decreases relative to 8 weeks at the center (24%) but is retained at the periphery. As a result, by 14 weeks, WT and SDC1-null corneas have similar axon density at the center but SDC1-null corneas continue to have significantly lower axon density at the periphery.

To determine whether the differences in axon density were impacted by their sex, axon density in female mice was assessed at 4 and 8 weeks. No sex-specific differences are observed; both 8-week male and female SDC1 corneas show a reduction in axon density relative to WT.

### The ICNs in SDC1-Null Corneas consist of Thicker Fibers Than Those in WT Corneas at 8w but ICNs in Both Genotypes of Mice Become Thinner With Age

Our ability to quantify ICN density in older male and female mice is compromised by three factors: (1) an increase in background staining of βIII tubulin within the epithelium, (2) thinning of ICNs, and (3) appearance of focal regions where ICN staining is too faint to assess. These factors, seen in both WT and SDC1-null 16-week male and female corneas (Fig. 1A), are also found in 40-week (10-month) female mice (Supplementary Fig. S4). To quantify differences in ICNs with aging, we quantify axon thickness at the center and periphery of WT and SDC1-null corneas as a function of age after birth from 5 to 40 weeks. During development, individual axons associate with one another via cell: cell adhesion molecules like NCAM and L1CAM to form parallel bundles—a process that is referred to as fasciculation; thicker bundles have more axons. After injury and in neuropathy, nerve fiber thickness decreases—a process that is referred to as defasciculation.^21,53^ The ICNs visualized in Figure 1A show a range of thickness, which corresponds to differences in the numbers of individual axons present in each fiber.

Axon thickness was quantified at the center and periphery in corneas from male WT and SDC1-null mice at 5, 8, and 16 weeks (4 months), and in female mice at 40 weeks (10 months). Data for axon thickness at the center and periphery are combined and mean thickness data are presented in Figure 1C. At 5 weeks of age, axon thickness is the same in both genotypes. At 8 weeks, when mean axon density is 17% to 37% less in SDC1-null corneas, mean axon thickness is 0.22 μm greater than WT—a significant increase of 14%. After 8 weeks, both WT and SDC1-null corneas lose axon thickness. At 16 weeks, ICNs have defasciculated in both WT (11%) and SDC1-null (12%) corneas relative to 8 weeks; axon thickness remains greater for SDC1-null mice. By 40 weeks, ICNs have defasciculated 19% in WT and 39% in SDC1-null corneas relative to 8 weeks; SDC1-null ICNs are thinner than those of WT. In addition, the number of SDC1-null corneas lacking a vortex increase to 50% (*n* = 4) at 40 weeks, whereas all of the of WT corneas (*n* = 5) retain their vortex.

### INTs and the Localization of LAMP1 are Reduced in Unwounded SDC1-Null Corneas

Assessing axon density and thickness in en face images fails to differentiate INTs, which extend apically at 90° angles from subbasal nerves (SBNs). To determine whether SDC1-null SBNs branch and extend INTs apically, βIII tubulin+ and GAP43+ INTs were visualized in 8-week adult control corneas using high-resolution 3D confocal images rotated to generate cross sections. GAP43 localization within axons and at axon growth cones is frequently used as a surrogate for sensory axon regeneration.^54–56^ Representative images showing cross-sectional 3D stacks are presented in Figure 2 either as projection images through 135 μm of corneal epithelial tissue (Fig. 2A) or as 0.5-μm slices (Fig. 2B). Figures 2A and 2B show localization of βIII tubulin and GAP43; Figure 2B also shows localization of LAMP1, a lysosomal marker.

Using ImageJ, INTs from images in Figure 2A were quantified at two sites above the SBNs (apical and basal). Data show significantly fewer INTs (50%) at both sites in SDC1-null corneas compared with WT corneas.

During homeostasis, fragments of ICNs shed due to damage or age are phagocytosed and degraded within the lysosomes of corneal epithelial cells.^9^ In Figure 2B, single (0.5-μm) 3D cross-sectional images are shown. The regions indicated by asterisks have been enlarged 3-fold to highlight sites of LAMP1 and ICN co-localization. Using ImageJ, mean pixel intensities for LAMP1 were also quantified at two sites (apical and basal) above the basement membrane zone. LAMP1 is reduced within the basal most cell layers in both genotypes. In addition, LAMP1 is reduced by more than 75% in both apical and basal cell layers in SDC1-null corneas.

In summary, while ICN density assessed by Sholl analysis is lower in SDC1-null mice at 8 weeks of age, ICN thickness is greater. Although 90% of SDC1-null corneas have ICNs that form a vortex by 5 weeks, 3 weeks later (8 weeks) only 72% of their corneas have a vortex. Syndecan-1–null corneas have fewer INTs extending apically from their SBNs. Reduced axon and INT density do not appear to result from increased degradation of ICNs by corneal epithelial cells because LAMP1, a marker for lysosomes, is reduced in SDC1-null epithelial cells. The increase in the mean thickness of SDC1-null axons and the instability of the vortex at the corneal center between 5 and 8 weeks are consistent with a loss of thinner axons. Both genotypes of mice lose ICN density and thickness as they age. After 14 weeks, quantification of axon density by Sholl analysis is compromised by reduced axon thickness coupled with increased background staining for βIII tubulin within corneas. By 40 weeks, ICNs are similar in thickness to those seen at 5 weeks after birth in both genotypes.

### In the Corneal Epithelium, mRNA and Protein for SDC3 are Increased and mRNA for SDC2 and SDC4 are Reduced and/or not Altered

Syndecan-1 is expressed primarily in epithelial cells, whereas SDC3 is expressed more in nerves. Syndecan-2 and -4 are expressed more in mesenchymally derived tissues and cell types. To determine whether other SDC family members compensate for the lack of SDC1 in the corneal epithelium, qPCR was performed for SDC1, SDC2, SDC3, and SDC4 on RNA extracts from the corneal epithelium harvested by debridement from WT and SDC1-null mouse corneas; data are shown in Figure 3A. Data show SDC3 RNA levels are increased 1.6-fold in SDC1-null corneal epithelium, but SDC2 and SDC4 RNA levels are decreased to levels 0.6- and 0.5-fold, respectively, compared with those in WT corneas.

The high expression of SDC1 in corneal epithelial basal and suprabasal cells^34^ makes it impossible to determine whether control or wounded corneal ICNs express SDC1 (data not shown). Both stromal nerves and ICNs express SDC3 (asterisks; Fig. 3B) in WT and SDC1-null corneas; SDC3 expression reduces dramatically as the nerves exit the stroma and enter the epithelial compartment (arrows; Fig. 3B). While the differences are subtle, SDC3 can be observed localized within ICNs in SDC1-null but not WT corneas consistent with elevated RNA level for SDC3 in the corneal epithelium. Low levels of expression of SDC2 and SDC4 within the corneal epithelium make it impossible to determine whether SDC2 and SDC4 proteoglycans are reduced in expression in SDC1-null compared with WT corneas (data not shown). Our data suggests partial compensation by SDC3 for the loss of SDC1 in the corneal ICNs but no upregulation of SDC2 and SDC4.

### ICNs Reinnervate Similarly in WT and SDC1-Null Corneas After 1.5-mm Debridement Wounds

Given that adult SDC1-null corneas have reduced ICN and INT density coupled with increased ICN thickness and fragility indicated by the loss of the vortex in 25% of the corneas between 5 and 8 weeks of age, it is not clear how well their ICNs will reinnervate their corneas after injury. Wild-type and SDC1-null mice were subjected to 1.5-mm debridement injuries and allowed to recover for varying times (1, 2, 3, 4, 7, 14, and 28 days); corneas were used for whole-mount confocal imaging to determine axon density. Panels showing data for WT and SDC1-null mice are in Supplementary Figures S8 and S9, respectively. The numbers of corneas used per time point are shown in Table 1 and representative images for WT and SDC1-null corneas 1 and 28 days after debridement are shown in Figure 4A; axon densities at the center and periphery are shown in Figure 4B. Because unwounded SDC1-null mice have significantly lower axon density in the center and periphery than WT mice (see Fig. 1B), axon density data for each genotype are normalized against those of unwounded mice.

Debridement wounds remove all of the ICNs within the wound area; reinnervation begins during re-epithelialization, which is complete within 20 to 22 hours in WT mice and 22 to 26 hours in SDC1-null mice.^34^ In the SDC1-null corneal center, axons rapidly extend between 1 and 2 days and between 3 and 4 days but these early attempts at reinnervation are transient and, at 7 days, axon density is the same as it was at 1 day. However, by 14 days in the center of SDC1-null corneas, a greater than 2-fold increase in axon density occurs relative to 7 days and is significantly greater than 1 day; the increased axon density is stable and maintained at 28 days. Despite improving over time, axon density at the center is lower than unwounded SDC1-null corneas at all time points assessed. In SDC1-null corneas at the periphery, axon density does not change significantly relative to controls at any time point.

Although no significant differences in normalized axon density are seen between genotypes at the center or periphery at any time point, differences in the timing of reinnervation between time points are seen. In WT mice, at the cornea center, ICN density stays the same between 1 and 2 days, increases between 2 and 3 days, and remains the same at 3, 4, and 7 days. Reinnervation between 1 and 4 days in WT corneas is retained unlike that seen in SDC1-null corneas. Between 7 and 14 days, ICN density increases dramatically and at 14 days is similar to controls. At 28 days, after erosions have formed,^15^ axon density at the center drops to levels below controls and becomes equal to that seen in SDC1-null corneas. In WT corneas, axon density at the periphery is less than controls for the first 3 days after debridement wounding; this is in contrast to SDC1-null corneas where ICN density is retained at the periphery at all time points. Axon density increases in the periphery in WT mice to control levels between 4 and 28 days. Data show that reinnervation of ICNs at the corneal center and periphery after debridement wounds is not significantly delayed in SDC1-null compared with WT mice.

### ICNs Defasciculate Transiently After Debridement but Recover Their Thickness by 28 Days in Both Genotypes

Next, axon thickness at 2, 3, 14, and 28 days after debridement injury was assessed at the center and periphery of WT and SDC1-null corneas (Fig. 4C). These studies are, to our knowledge, the first to quantify ICN loss of axon thickness (defasciculation) in response to debridement injury. In WT mice, normalized axon thickness at the center is significantly less than controls at 2, 3, and 14 days but is similar to controls at 28 days. In the periphery, normalized WT ICN thickness is lower than control at 2 and 14 days, but is similar to controls at 3 and 28 days. Normalized axon thickness is less than controls at the center and periphery of SDC1-null corneas at 2 and 3 days but similar to controls at 14 and 28 days. At the center, normalized axon thickness in SDC1-null corneas is less than WT at 2 days; at the periphery, it is less than WT at 3 days. However, later times points show no significant differences in normalized axon thickness in SDC1-null corneas compared with unwounded controls. Despite the fact that neither genotype recovers control levels of axon density after debridement, both restore axon thickness after debridement.

### The ICNs in SDC1-Null Corneas Reinnervate Slower and Defasciculate More Than WT After 1.5-mm Trephine Wounds

After 1.5-mm trephine wounds, 50% of the ICNs are crushed or severed. Axonal debris is removed by corneal epithelial cell phagocytosis. During recovery, elongating ICNs extend between and under stationary corneal epithelial cells that remain adherent to the basement membrane. Panels showing the data from individual corneas for WT male and female mice after trephine injury are in Supplementary Figures S5 and S6; Supplementary Figure S7 shows images of SDC1-null corneas. Female WT mice were also used to determine the rate of recovery of the ICNs after trephine injuries; because no sex-specific differences were observed (data not shown), male SDC1-null mice were used for the wound healing studies presented below and data compared with age-matched male WT mice.

To determine whether the ICNs of SDC1-null mice reinnervate trephine wounds similar to WT, corneas were subjected to 1.5-mm trephine injuries and allowed to recover for varying times (1, 2, 3, 4, 7, 14, 28, and 42 days) and used for whole-mount confocal imaging to determine axon density. The numbers of corneas used per time point are shown in Table 1 and representative images for 1, 28, and 42 days are shown in Figure 5A. In both genotypes, at 1 day after trephine injury, loss of ICNs at the center is observed along with partial retention of the vortex; over time, regions where ICNs are missing become less pronounced. The number of SDC1-null corneas lacking a vortex remains consistent over time after trephine injury. Axon densities at the center and periphery for these and all other time points were quantified and data are shown in Figure 5B. Because unwounded SDC1-null mice have significantly lower axon density in the center and periphery than WT mice (see Fig. 1B), axon density data are normalized against those of unwounded mice for each genotype and anatomic location (center or periphery). For the 42-day time point, unwounded age and genotype-matched controls are used to normalize axon density data.

In SDC1-null mice, by 1 day after trephination, axon density in the center is reduced to slightly less than 50%—a value similar to that seen in WT mice indicating that the extent of axon damage induced by injury is similar in both genotypes. Axon density in SDC1-null corneas at the center is lower than controls at 1 to 4 days after wounding. While axon density at the center of SDC1-null mice is reduced compared with unwounded mice at 28 days after injury, by 42 days, axon density is restored to levels similar to unwounded age-matched controls. By contrast, in WT mice, axon density is restored to levels similar to controls by 4 days.^14^ In the periphery, no significant decrease in axon density is observed at any time point for SDC1-null corneas; by contrast, at 2 days, ICN density in WT corneas is reduced relative to controls and SDC1-null corneas. Axon density recovers significantly slower after trephine injury in SDC1-null mice.

### ICNs in SDC1-Null Corneas Defasciculate After Trephine Injury

Changes in axon thickness (defasciculation) were also assessed at the center and periphery at 3, 14, 28, and 42 days in WT and SDC1-null corneas (Fig. 5C). Because axon thickness is significantly different between genotypes of adult mice prior to injury (see Fig. 1C), data are expressed relative to control axon thickness for each genotype. Values less than 1 indicate reduced axon thickness compared with unwounded controls. In SDC1-null corneas at 3, 14, and 28 days in the center and periphery, axon thickness is decreased relative to unwounded controls. In WT corneas, axon thickness is decreased relative to controls at the center only at 28 days. By 42 days after trephine injury, axon thickness in SDC1-null corneas is greater than in age-matched controls at both the center and periphery. In WT corneas, 42 days after trephine injury axon thickness is greater in the periphery but not at the corneal center. These data show that in trephine wounded SDC1-null corneas, ICNs remain defasciculated longer compared with WT mice.

### The Numbers of GAP43+βIII tubulin+ INTs Projecting Apically Increase After Trephine Injury in SDC1-Null Corneas

In Figure 2A, we show that there are approximately 50% fewer apically projecting INTs in SDC1-null corneas compared with WT corneas. To determine the timing and extent of reinnervation of the INTs high-resolution 3D confocal images were acquired of trephine-injured WT and SDC1-null corneas stained to localize βIII tubulin and GAP43; confocal image stacks were rotated to reveal cross-sectional images projecting 135 μm through the tissue. The images presented in Figure 6 are obtained near the corneal center at 4, 14, and 28 days after trephine wounds in WT and SDC1-null mice. At 14 days after trephine injury, GAP43 staining reveals increased punctate staining suggesting that INTs are being turned over. βIII tubulin+ and GAP43+ INTs were quantified and data presented are normalized relative to the numbers of INTs present in unwounded corneas. In SDC1-null corneas, βIII tubulin+ INTs are increased more than 3-fold relative to unwounded corneas at all three time points assessed; GAP43+ INTs are also increased in SDC1-null corneas but the extent of increase is less than that seen for βIII tubulin+INTs. By contrast, in WT corneas, βIII tubulin+ and GAP43+ INTs are the same or reduced relative to controls at all three time points assessed.

### LAMP1 Increases in SDC1-Null but not WT Corneas After Trephine Injury

Using single 0.5-μm sections through 3D confocal image stacks, LAMP1+ lysosomes and βIII tubulin+ and GAP43+ INTs are visualized and quantified in WT and SDC1-null corneas allowed to heal for 14 days after trephination (Fig. 7). When normalized relative to unwounded corneas (Fig. 2B), LAMP1 increases more than 3-fold in apical-most cell layers in SDC1-null corneas at 14 days after trephine injury. By contrast, in WT mice, there is no change in the amount of LAMP1 relative to controls after trephine wounding. These data show that stable reinnervation of the ICNs and INTs after 1.5-mm trephine injury in WT mice does not require increased numbers of LAMP1+ lysosomes. Syndecan-1–null corneal epithelial cells have fewer LAMP1+ lysosomes during homeostasis; trephine injury increases lysosomal numbers significantly. Consistent with increased axonal turnover after injury, localization of LAMP1 with GAP43 and βIII tubulin increase after trephine injury compared with unwounded controls (Fig. 2B).

### SDC1-Null Corneal Epithelial Cells and Epidermal Keratinocytes are Less Efficient at Phagocytosing Opsonized Particles Than WT Cells

Reinnervation after trephine wounds requires corneal epithelial cells to phagocytose debris generated by the degeneration of 50% of the ICNs (SBNs and INTs) at the corneal center. After debridement wounds, corneal epithelial cells and INTs are removed; during reinnervation axons extend between migrating corneal epithelial cells. The delay in recovery of axon density and thickness in SDC1-null corneas after trephine but not debridement wounds suggests that phagocytosis of debris by SDC1-null corneal epithelial cells may be impaired. The increase in the numbers of LAMP1+ lysosomes in response to trephine injury in SDC1-null but not WT corneal epithelial cells also suggests altered lysosomal function in SDC1-null corneal epithelial cells after partial denervation by trephination.

To determine if phagocytosis differs between genotypes, WT and SDC1-null corneal epithelial cells were generated from explants; equal numbers of opsonized fluorescently labeled 1-μm latex beads were added to cultures. After 24 hours, cells were washed and fixed and fluorescent beads within cells quantified. Syndecan-1–null corneal epithelial cells phagocytose 50% fewer beads compared with WT corneal epithelial cells (Fig. 8). To determine whether epidermal keratinocytes lacking SDC1 also show differences in phagocytosis compared with WT, uptake of fluorescently labeled beads was assessed in these cells as well; inhibitors (CQ and BafA1) were included in the assays. Data show that SDC1-null epidermal keratinocytes, like corneal epithelial cells, phagocytose 50% fewer beads than WT cells. Chloroquine and BafA1 reduce phagocytosis to 30% control levels in WT cells; while CQ and BafA1 also inhibit phagocytosis in SDC1-null keratinocytes, BafA1 inhibits bead uptake more than CQ. Experiments were repeated allowing beads to incubate with explants for 1 hour and the results obtained were similar to those obtained after 24 hours (data not shown).

## Discussion

### Morphologic Differences are Seen Between WT and SDC1-Null ICNs in Adults and With Aging

These studies demonstrate the important role played by SDC1 in ICN homeostasis and highlight the importance of phagocytosis of axonal debris by corneal epithelial cells in the reinnervation of ICNs after injury. Differences in the ICN density are seen between WT and SDC1-null mice after the eyelids open but by 4 and 5 weeks after birth, WT and SDC1-null corneas have similar axon densities and thickness and both genotypes and sexes show well-formed vortices at their corneal centers. By 8 weeks of age, male and female SDC1-null mice have significantly reduced axon density compared with WT mice at both the corneal center and periphery. At the center, 8-week male and female SDC1-null mice have 17% and 31% (average 24%) fewer axons than WT mice, whereas at the periphery, male and female SDC1-null mice have 38% and 37% (average 37.5%) fewer axons. The reduction in axon density in SDC1-null corneas is accompanied by reduced localization of LAMP1 in corneal epithelial cells and reduced βIII tubulin and GAP43 within the INTs.

While none of the 5-week SDC1-null corneas lacked a vortex, 25% to 30% of 8-week SDC1-null mice lacked an ICN vortex compared with less than 10% in WT mice. The percentage of SDC1-null corneas lacking a vortex is not altered after trephine wounding. The formation of a spiral vortex at the corneal center is believed to occur randomly during development and is regulated stochastically.^14^ It has no known function. The axons at the corneal center are further away from the nerve cell body that gave rise to them compared with axons at the periphery; the cells that provide support for the axons of the vortex are among the most differentiated of the epithelial basal cells of the cornea because they differentiated from progenitor cells at the corneal limbus. When denervated corneas are placed in organ culture, vortex axons at the center degenerate before those at the periphery suggesting that they are more fragile.^57^ These features likely contribute to the loss of the vortex in SDC1-null corneas between 5 and 8 weeks of age.

Between 8 and 14 weeks after birth, SDC1-null corneas retain 100% of their ICN density at the periphery while losing 23% at the center. By contrast, between 8 and 14 weeks, WT mice lose 23% of their axon density at the periphery and 41% at the center. The loss of axons due to aging between 8 and 14 weeks is delayed in SDC1-null mice compared with WT mice.

Fasciculation of ICNs, assessed by quantifying axon thickness, increases between 5 and 8 weeks in both genotypes with SDC1-null ICNs becoming significantly thicker (13%) than WT ICNs by 8 weeks. Thicker bundles of ICNs can form more integrin: ECM adhesions with corneal epithelial cells; more axons within each bundle favors their stabilization via cell: cell adhesion mediated by NCAM and L1CAM and thicker fibers transmit electrical impulses faster enhancing responses to heat, cold, and pain.^21,22^ Differences in fasciculation of ICNs in mice lacking SDC1 are consistent with data showing the importance of HSPGs in mediating axon fasciculation in drosophila^58^ and with the roles played by the glycopolymer polysialic acid in corneal sensory nerve fasciculation in chick.^59^ Whether this increased thickness is due to fragility of thinner SDC1-null axons, to their reduced ability to branch and extend apically and become INTs, or to other causes is not clear. Differences in ICN thickness between genotypes are lost during aging. In SDC1-null corneas, ICN thickness drops 40% between 8 and 40 weeks of age; by contrast, WT corneal ICN thickness drops 19% between 8 and 40 weeks of age.

### Induction of SDC1-Null Corneal Epithelial Cell Migration by Debridement Injury Attenuates Differences in ICN Reinnervation Between Genotypes

While SDC1 expression is increased in the injured hypoglossal nerve in mice,^60^ we have not been able to demonstrate SDC1 within ICNs in control or wounded WT corneas (data not shown) due to high expression of SDC1 within corneal epithelial cells. Axon density takes significantly longer to recover and axons remain defasciculated longer after trephine but not after debridement injury in SDC1-null mice. The injury response of the cornea to debridement wounding has been well studied.^57,61^ After debridement, corneal epithelial cells increase their rate of protein synthesis, reduce their adhesion to one another to allow leading edge extension, and reduce adhesion to the substrate to allow migration and sliding of the cell sheet to close the wound. The ICNs embedded within the migrating epithelial sheet extend toward the leading edge as the sheet migrates. Here, we show that the ICNs in WT and SDC1-null corneas partially defasciculate and become thinner during sheet movement as they elongate; they then begin to get thicker after migration is completed. As wound edges merge, cell proliferation occurs and cell:cell and cell:substrate adhesions attempt to reform to restore barrier and sheet integrity. Spontaneous recurrent corneal erosions begin to form in mice 14 to 28 days after debridement wounding. Erosion formation correlates with persistently elevated cell proliferation in the corneal periphery,^34^ withdrawal of epithelial cells from the cell cycle near the center of the cornea where erosions typically form, and, finally, with apoptosis of corneal epithelial cells within erosion sites.^14^ Using topically applied Mitomycin C (MMC), ICN recovery after debridement is enhanced and erosions reduced.^62^ It remains to be seen whether MMC treatment impacts the reinnervation of the ICNs after trephine injury.

Little is known about how corneal epithelial cells respond to partial denervation induced by trephination.^14^ A dulled 1.5-mm trephine is placed on the corneal surface and rotated gently to crush or sever the SBNs under the trephine blade resulting in the loss of 50% of the total axon density in the central cornea within 24 hours of injury. The corneal epithelium is left in place but epithelial cells under the blade are crushed. The few cells killed are phagocytosed by their neighbors, basal cells proliferate, apical tight junction barrier reforms, and typically, the trephine mark is no longer visible within 48 hours after injury. Intraepithelial corneal nerves can target to the corneal center and send INTs apically in the absence of sheet movement or corneal epithelial cell migration. Here, we show that while SDC1-null ICNs are slower to reinnervate the cornea after trephine injury, they are more efficient than WT ICNs at extending INTs apically after trephine wounds. Axon targeting of INTs within the SDC1-null corneal epithelium is not defective after trephine injury. During homeostasis, there are 50% fewer INTs in the apical- and basal-most cell layers of the SDC1-null cornea. While all of the causes of reduced INT targeting in unwounded SDC1-null corneas are not known, differences in the numbers of LAMP1-positive lysosomes may play a role.

### Endocytosis of Opsonized Latex Beads is Impaired in SDC1-Null Epithelial Cells

Intraepithelial corneal nerves are simultaneously growing and shedding degenerating axon tips between corneal epithelial basal cells. Debris is opsonized and phagocytosed by the corneal epithelial cells. Cell surface–associated HSPGs are known to mediate the endocytosis of a variety of growth factor receptor complexes as well as morphogens and viruses.^63^ Corneal epithelial cells and epidermal keratinocytes from SDC1-null mice phagocytose approximately 50% fewer fluorescently labeled serum-opsonized 1-μm latex beads than WT cells. Phagocytosed particles are transported to the lysosomes where their contents are degraded; phagocytosed latex beads accumulate within cells in endosomes and lysosomes. Chloroquine and BafA1 reduce phagocytic and autophagic flux in cells.^64^ Both inhibitors reduce bead uptake in WT keratinocytes by approximately 60% to 70%. While BafA1 also blocks bead uptake in SDC1-null keratinocytes by 60% to 70%, CQ reduces it by only 30%. Why SDC1-null keratinocytes are partially resistant to CQ but not BafA1 is not clear and merits further study. Our results show that epithelial cells lacking SDC1 mediate phagocytosis poorly compared with WT cells; this defect likely contributes to the delay in reinnervation after trephine injuries.

A reduced ability of corneal epithelial cells to clear debris would be expected to contribute to pathology. Patients with malaria, lupus, and rheumatoid arthritis (RA) treated with CQ develop chloroquine keratopathy (CK). First described in the late 1950's by Hobbs and Calnan,^65^ CK was also seen in workers in factories making CQ.^66^ Studies of patients with CK show debris accumulating within the corneal epithelium and disruptions of the ICNs by in vivo confocal imaging.^67^ Chloroquine retinopathy is also observed in patients taking CQ long term due to reduced phagocytosis by RPE cells.^68^

Numerous corneal dystrophies have been described; they are estimated to impact as many as 2% of the general population.^69^ Many mutations lead to the accumulation of debris within or beneath the corneal epithelial basal cells. Some of these defects are likely due to the inability of mutated corneal epithelial cells to maintain the high rate of phagocytic flux needed to remove the shed tips of corneal ICNs. Because the unwounded adult SDC1-null cornea is clear and we see no evidence of debris accumulating over time, the reduction in corneal epithelial cell phagocytosis we observed is not sufficient to lead to pathology in unwounded adult mice.

We showed previously that the activation of SDC1-null epidermal and corneal epithelial cells is attenuated in response to skin and debridement injury to the cornea, increasing the time it takes for skin and cornea to re-epithelialize.^29,34^ The trephine wound studies presented here show that successful reinnervation of the ICNs at the corneal center in both genotypes of mice does not require corneal epithelial cell migration and activation. While reinnervation of the ICNs in SDC1-null mice is slower after trephine injury, this delay is eliminated by debridement injury, which removes axonal debris while activating corneal epithelial cell migration and reducing cell:cell, cell:substrate, and cell:ICN adhesion.

### After Trephine Injury, the INT Deficit Seen in Unwounded SDC1-Null Corneas is no Longer Present

GAP43 is expressed in the nervous system, plays an important role in forming and maintaining synaptic connections in the developing brain, and is upregulated as one of the regeneration associated genes during axonal regeneration after injury:^44,70,71^ GAP43 is also considered a marker for regenerating axons. It is reduced in expression in skin biopsies from patients with diabetic neuropathy^72,73^ but increased in expression in early-stage peripheral neuropathies.^56^ GAP43 was first demonstrated in the cornea by Martin and Bazan^45^ and is one of several RAGs genes whose mRNA expression increases 14 days after lamellar flap surgery.^46^

The numbers of βIII tubulin+GAP43+ INTs that extend apically are reduced in SDC1-null mice before wounding but after trephine injury, the numbers of INTs in SDC1-null corneas are increased compared with WT. This observation merits further study because the factors that regulate INT branching from the SBNs and targeting to the apical aspect of the corneal epithelium are not known. Corneal epithelial cells in WT mice may secrete factors that induce INTs to branch and elongate. Partial corneal denervation appears sufficient to enhance targeting of INTs apically in SDC1-null mice making them a good model to use for studies of axon targeting.

## Conclusions

Mice lacking SDC1 are viable and fertile.^34^ Their phenotype, when stressed, reveals tissue-specific gain and loss of function attributes^28^ that have advanced our understanding of numerous complex pathologies including cardiovascular disease,^74–76^ inflammatory conditions,^77–79^ cancer,^80–82^ and wound healing.^29,34,83^ The SDC1-null mouse is used here to study PNS reinnervation using corneal injury models. Table 2 summarizes the changes that are observed in the ICNs and the corneal epithelium in mice lacking SDC1 during homeostasis and after injury. Data show that SDC1 plays important role in axon stability, branching, and targeting of INTs to apical-most cell layers, as well as fasciculation and defasciculation during postnatal development, with aging, and in response to injuries that sever axons.

**Table 2:**
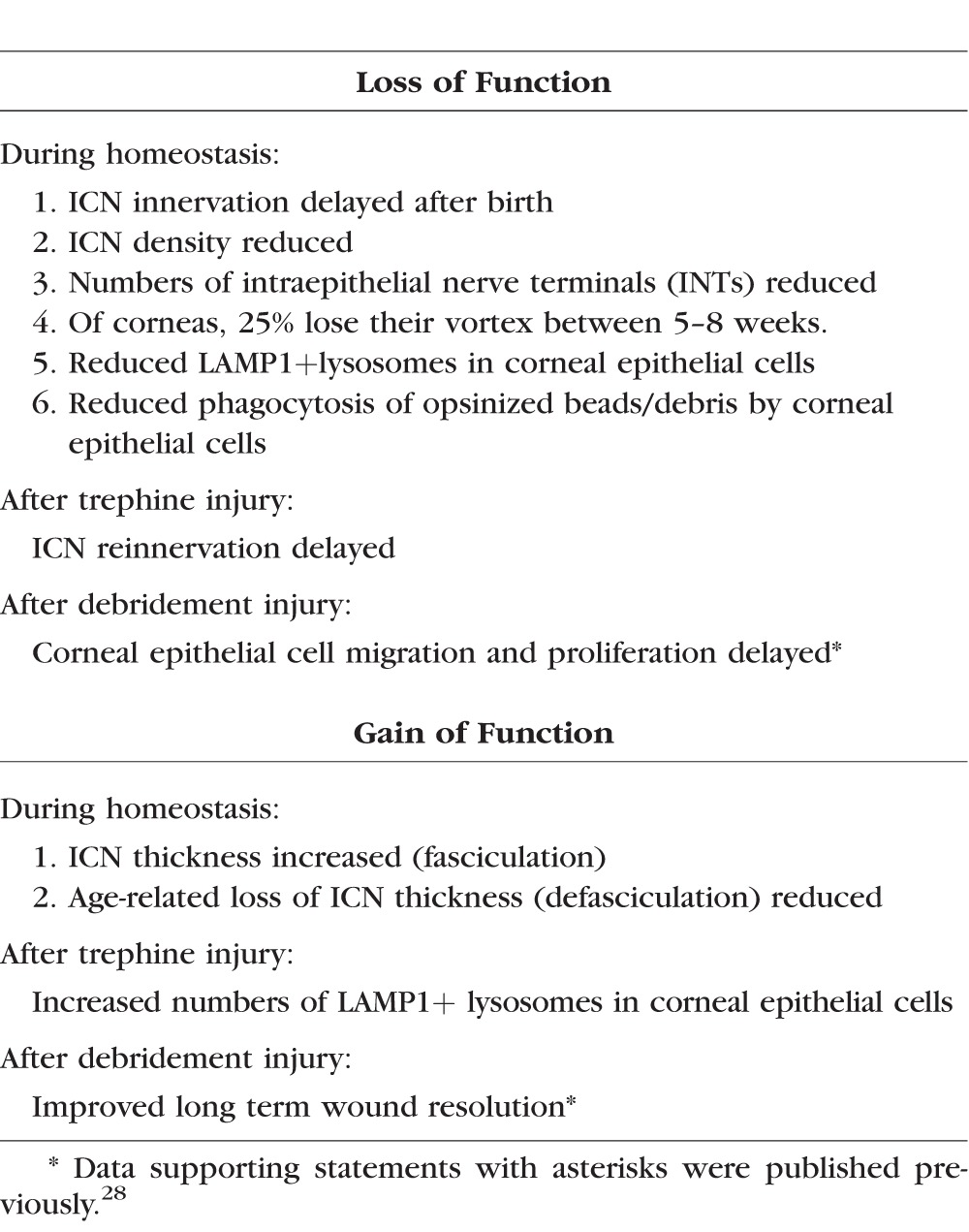
Impact of the Loss of Syndecan-1 on Corneal Epithelial Cells and ICNs*

Heparan sulfate proteoglycans regulate axon guidance and fasciculation during development.^18,32^ Studies by Liu and colleagues^84^ show that the lack of SDC1 on mouse mammary epithelial cells increases HS on other proteoglycans. Studies by Ramani and colleagues^85^ show that shedding of the SDC1 extracellular domain increases SDC1 core protein synthesis to maintain SDC1 on the cell surface. In vivo, when SDC1 shedding is induced by Staphylococcus aureus infections in the lung, it's cell surface expression assessed by flow cytometry is maintained presumably by cells increasing surface expression of SDC1 from intracellular vesicles.^86^ Taken together, data show that when cells that express SDC1 are stressed, they maintain cell surface HSPG expression by increasing synthesis of other HSPG core proteins. In SDC1-null corneal epithelial cells, mRNA for SDC3 but not SDC2 and SDC4 are upregulated; localization of the SDC3 core protein appears increased in SDC1-null ICNs but not in corneal epithelial cells. A detailed analysis of the HSPGs on other core proteins in SDC1-null cells and tissues would help gain insight into the roles HSPGs play in regulating axon targeting and fasciculation during axon regeneration and aging.

As the population in the Unites States ages, the number of people with small fiber neuropathy will increase.^43,87^ The cornea provides a well-characterized model system to better understand how sensory nerves are maintained and what happens to them when they are injured. As neurologists increase their use of in vivo confocal imaging for diagnosis of small fiber neuropathy,^10^ it is critical that vision scientists continue to increase our understanding of corneal SBNs and INTs. We hope that knowledge gained using the mouse cornea as a model system leads to new approaches that will minimize age-related sensory axon loss and to optimize axon recovery after injury.

## Supplementary Material

Supplement 1
