# Supplementary figures and images for: Alterations in Corneal Sensory Nerves During Homeostasis, Aging, and After Injury in Mice Lacking the Heparan Sulfate Proteoglycan Syndecan-1
Source: Invest Ophthalmol Vis Sci. 2017 Oct;58(12):4959–75. doi: 10.1167/iovs.17-21531 (PMC5627677; doi:10.1167/iovs.17-21531)

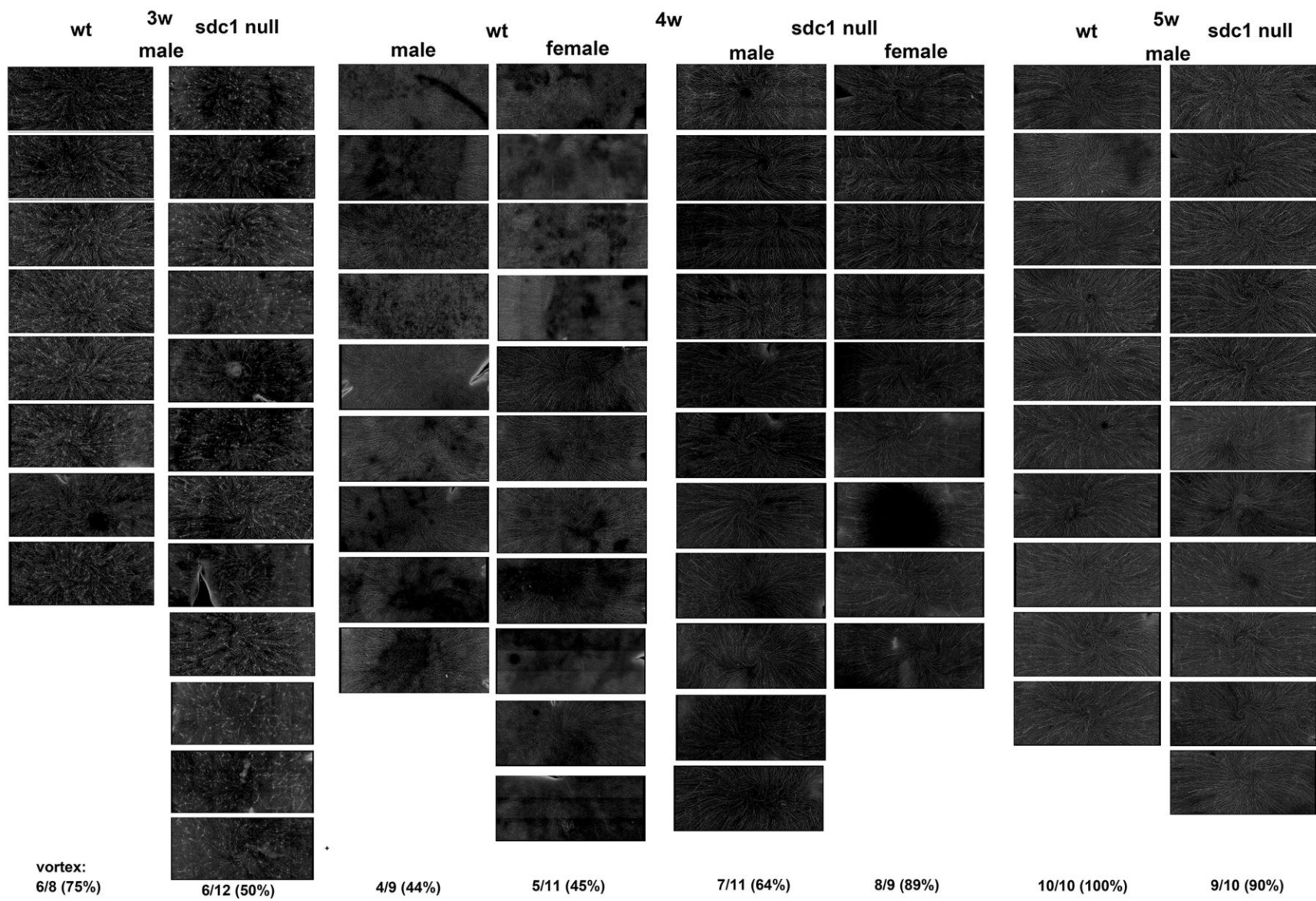

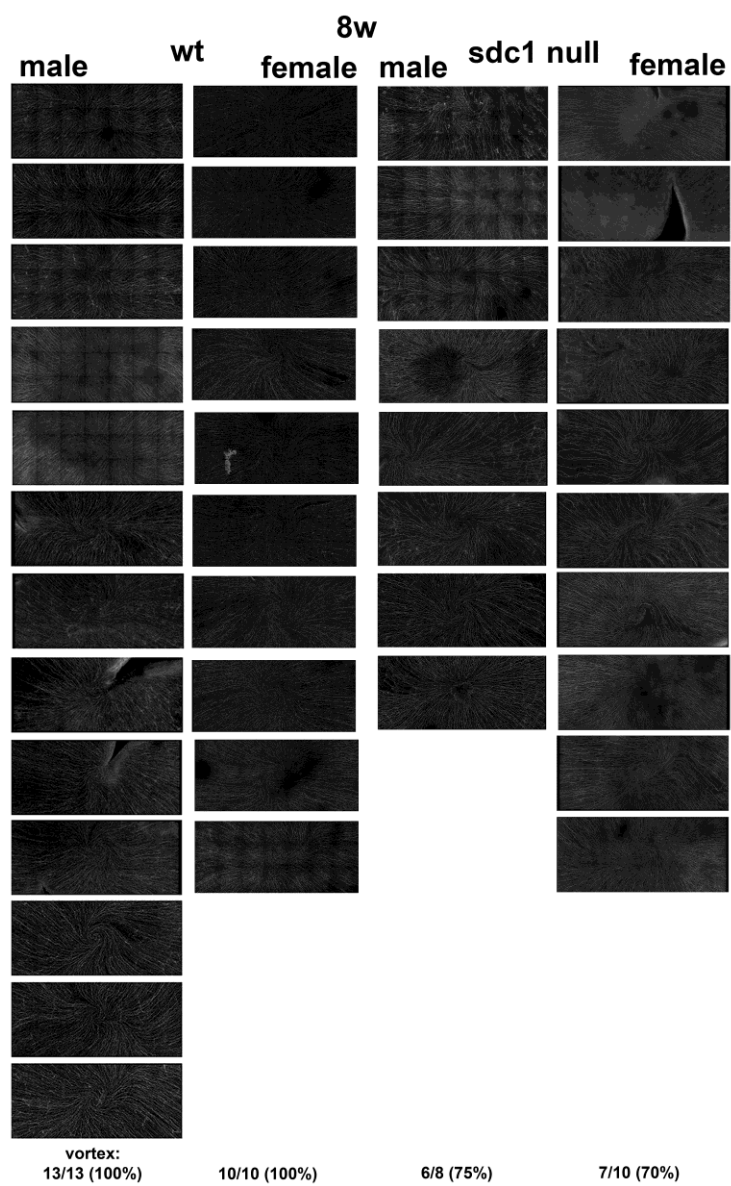

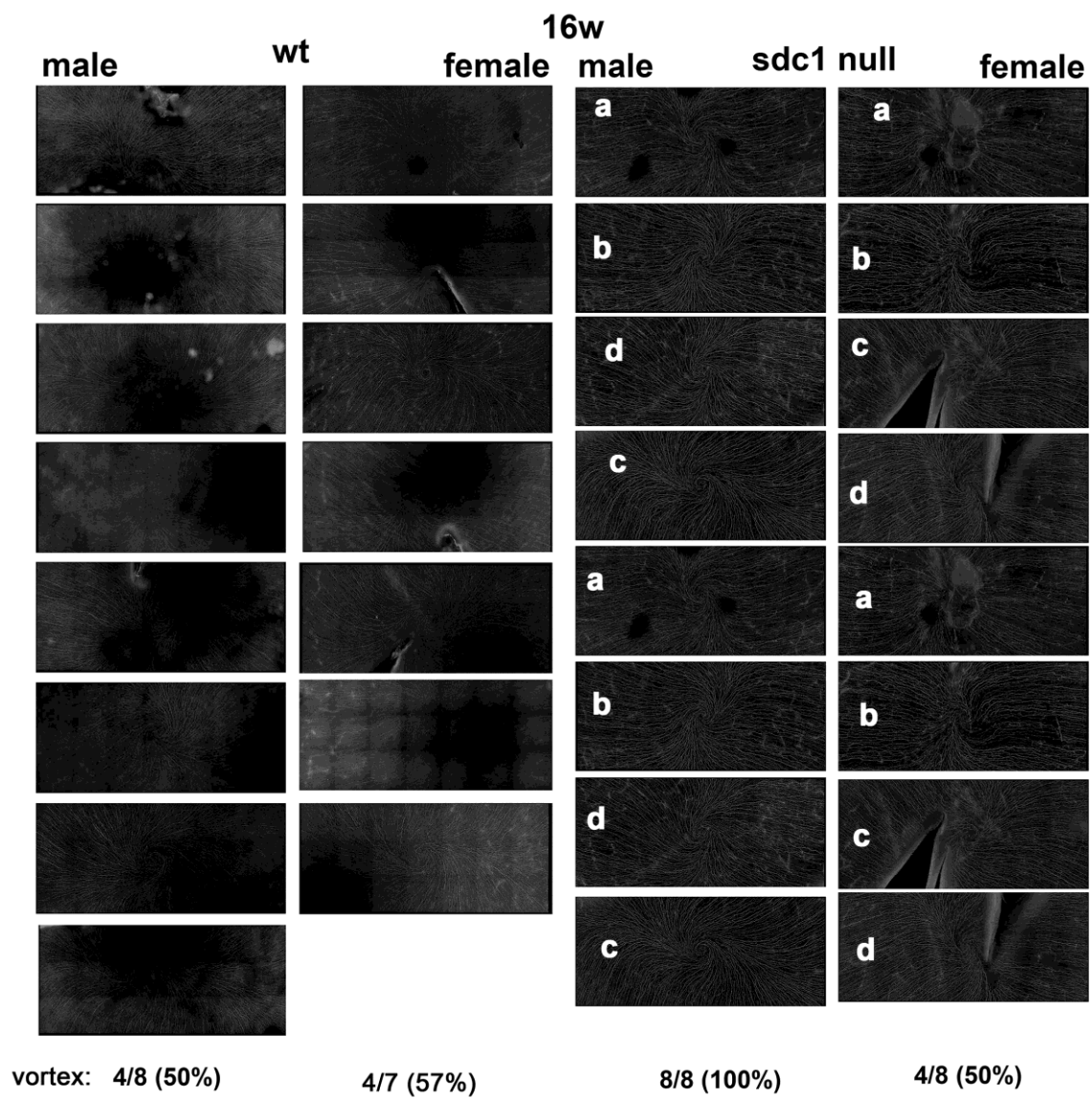

Supp. 3 16w unwounded

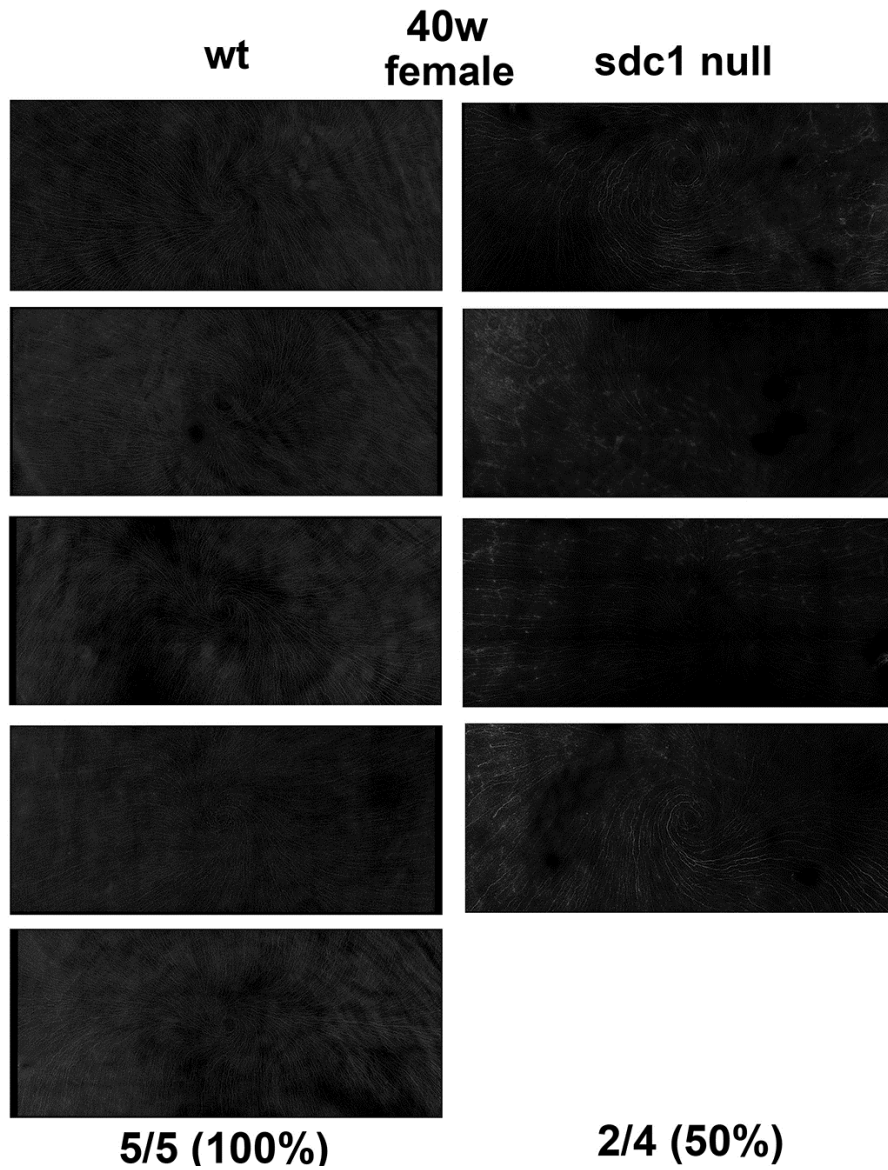

Supp. 4. 40w post natal dev

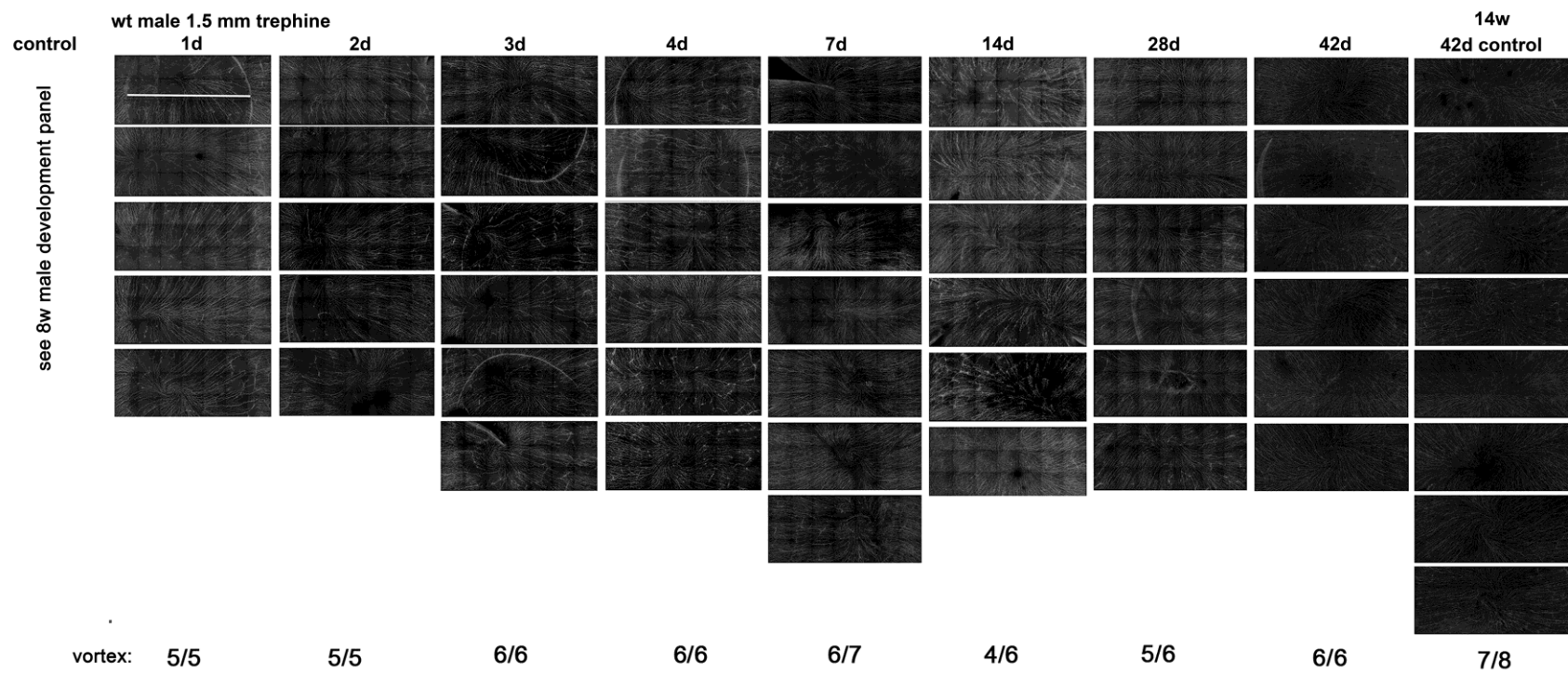

Supp. 5 wt male tre

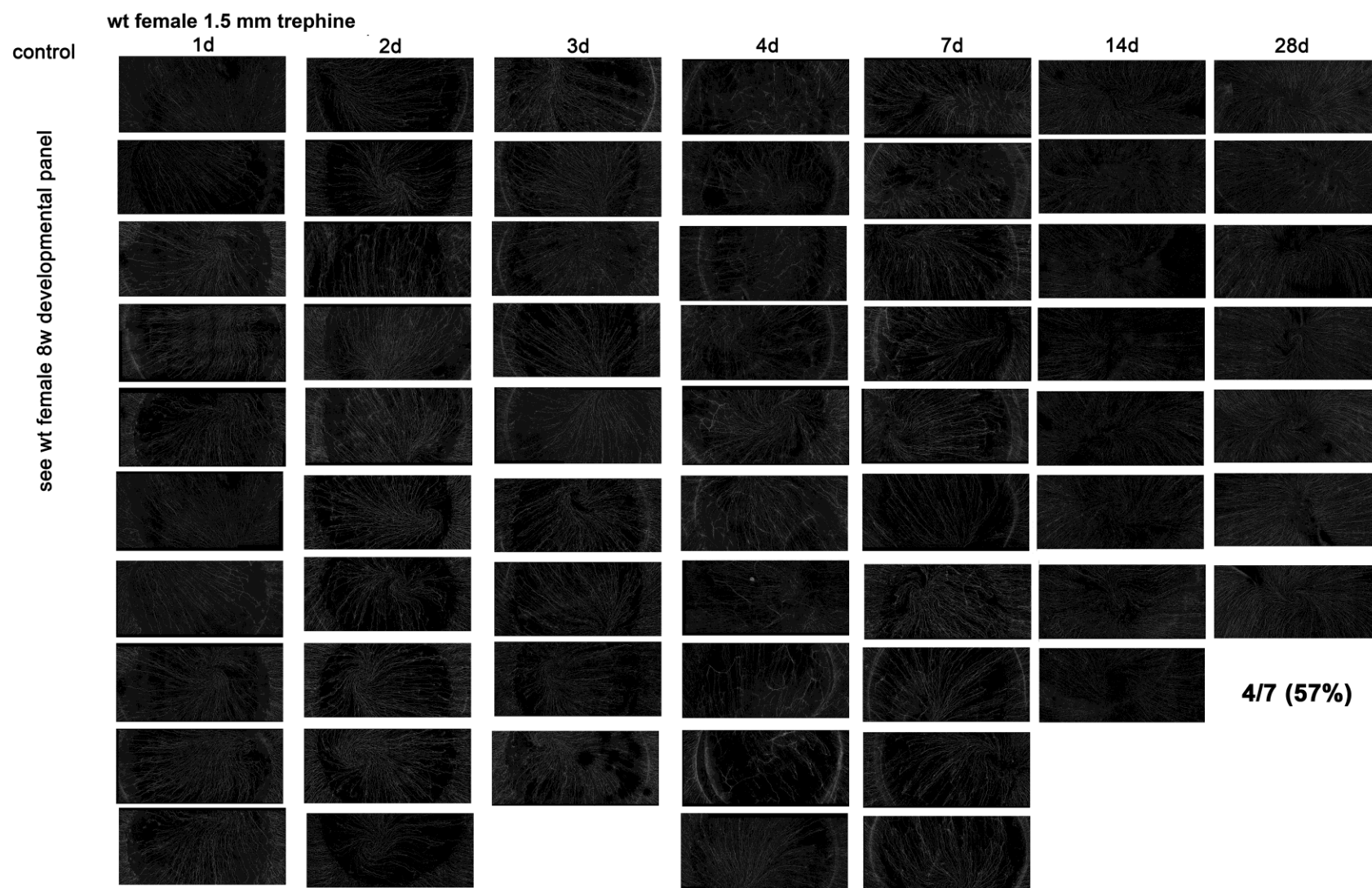

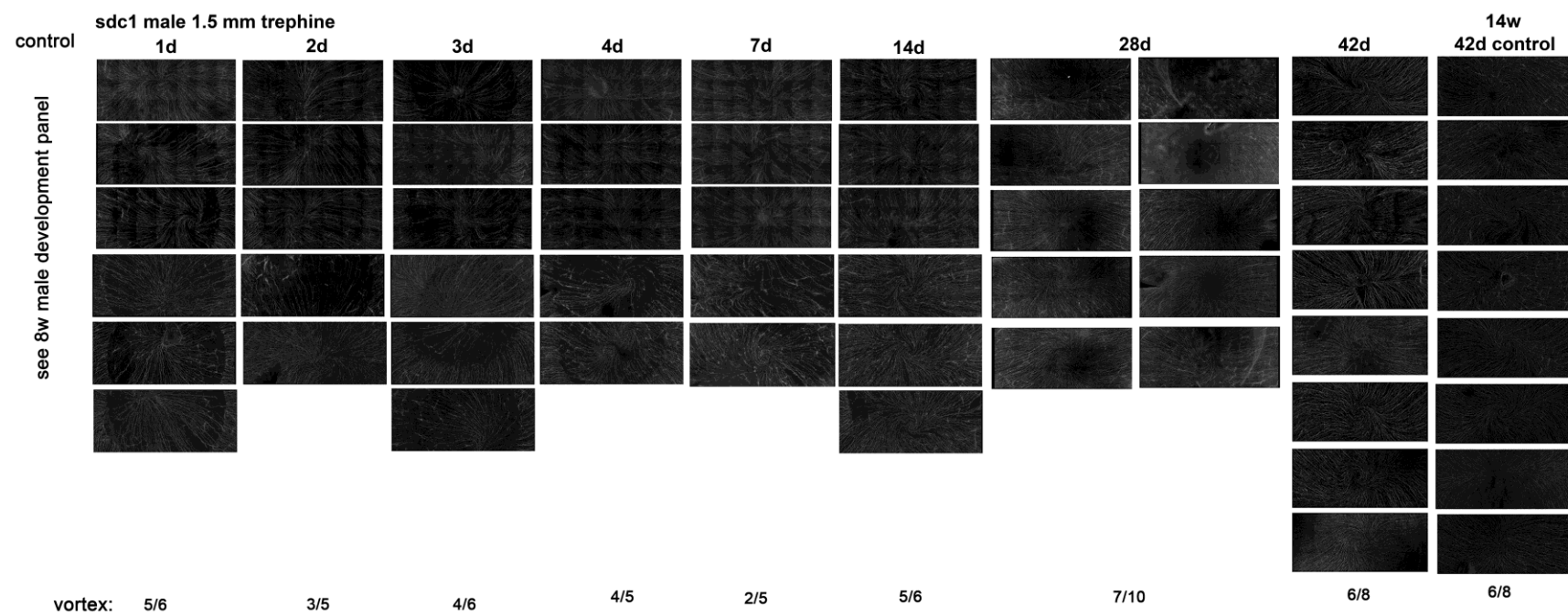

Supp. 7 sdc1 null tre

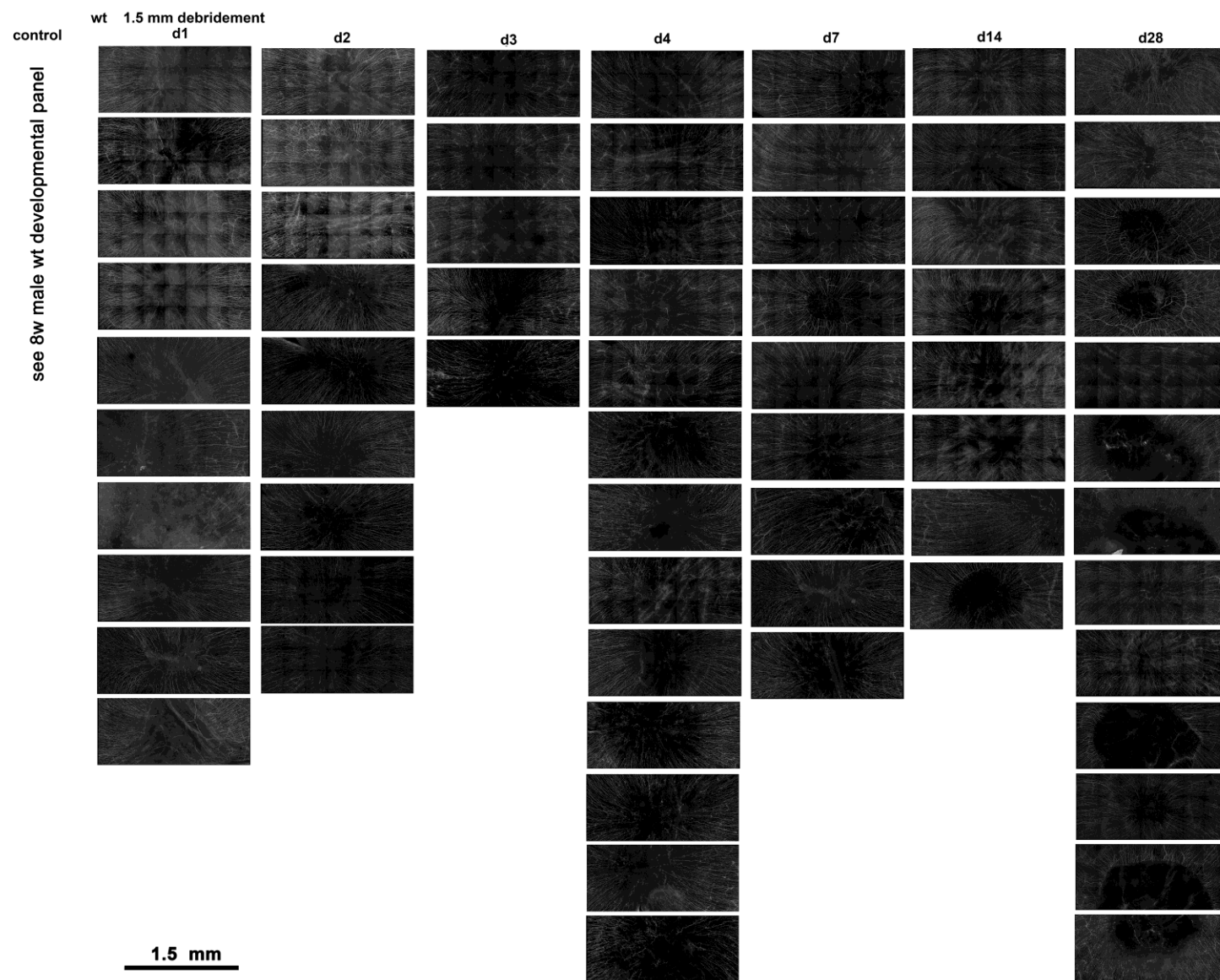

Supp. 8. wt sm

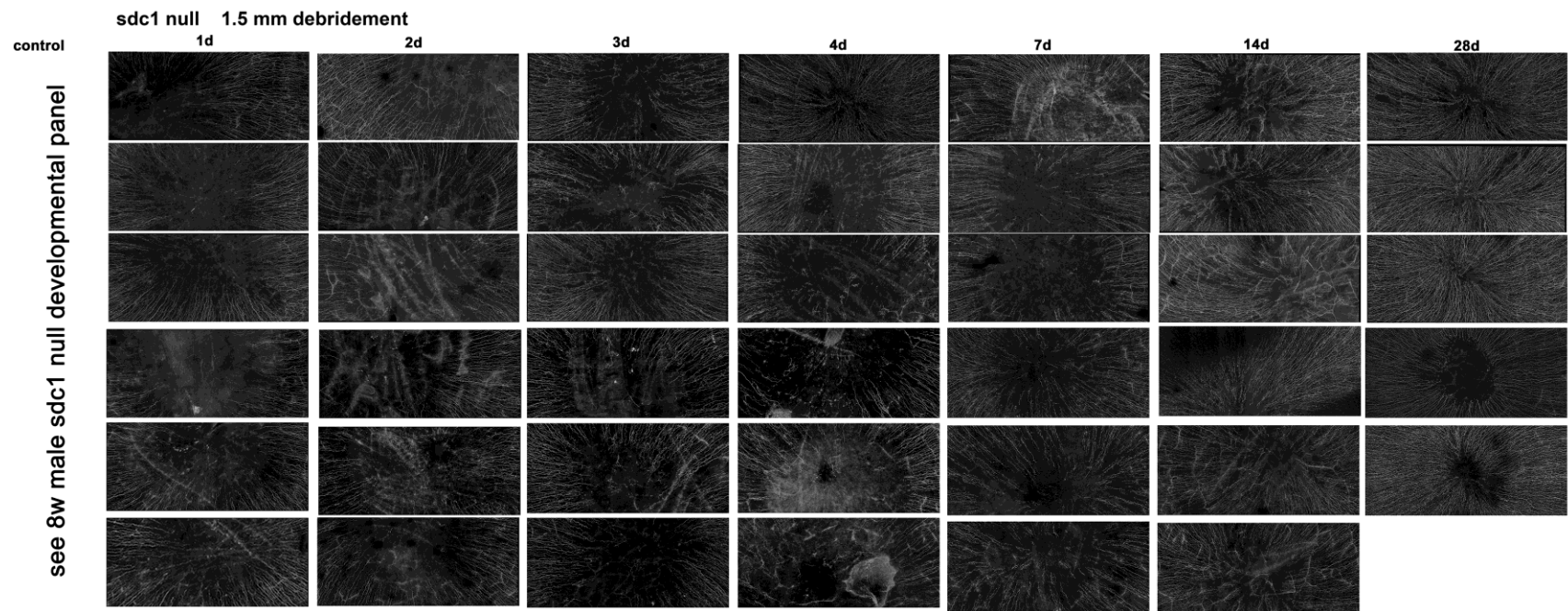

Supp. 9. sdc1 null sm

Supplement: Supplement 1 [file iovs-58-11-20_s01.pdf]
